# Physiological characterization of the wild almond *Prunus arabica* stem photosynthetic capability

**DOI:** 10.3389/fpls.2022.941504

**Published:** 2022-07-29

**Authors:** Taly Trainin, Hillel Brukental, Or Shapira, Ziv Attia, Vivekanand Tiwari, Kamel Hatib, Shira Gal, Hanita Zemach, Eduard Belausov, Dana Charuvi, Doron Holland, Tamar Azoulay-Shemer

**Affiliations:** ^1^Department of Fruit Tree Sciences, Volcani Center, Newe Ya'ar Research Center, Agricultural Research Organization, Ramat Yishay, Israel; ^2^Faculty of Agriculture, The Robert H. Smith Institute of Plant Sciences and Genetics in Agriculture, Hebrew University of Jerusalem, Rehovot, Israel; ^3^Volcani Center, Institute of Plant Sciences, Agricultural Research Organization, Rishon LeZion, Israel

**Keywords:** almond, CO_2_-assimilation, stem photosynthetic capabilities (SPC), stem recycling photosynthesis (SRP), stomata, transpiration, photosynthesis

## Abstract

Leaves are the major plant tissue for transpiration and carbon fixation in deciduous trees. In harsh habitats, atmospheric CO_2_ assimilation *via* stem photosynthesis is common, providing extra carbon gain to cope with the detrimental conditions. We studied two almond species, the commercial *Prunus dulcis* cultivar “Um-el-Fahem” and the rare wild *Prunus arabica*. Our study revealed two distinctive strategies for carbon gain in these almond species. While, in *P. dulcis*, leaves possess the major photosynthetic surface area, in *P. arabica*, green stems perform this function, in particular during the winter after leaf drop. These two species' anatomical and physiological comparisons show that *P. arabica* carries unique features that support stem gas exchange and high-gross photosynthetic rates *via* stem photosynthetic capabilities (SPC). On the other hand, *P. dulcis* stems contribute low gross photosynthesis levels, as they are designed solely for reassimilation of CO_2_ from respiration, which is termed stem recycling photosynthesis (SRP). Results show that (a) *P. arabica* stems are covered with a high density of sunken stomata, in contrast to the stomata on *P. dulcis* stems, which disappear under a thick peridermal (bark) layer by their second year of development. (b) *P. arabica* stems contain significantly higher levels of chlorophyll compartmentalized to a mesophyll-like, chloroplast-rich, parenchyma layer, in contrast to rounded-shape cells of *P. dulcis's* stem parenchyma. (c) Pulse amplitude-modulated (PAM) fluorometry of *P. arabica* and *P. dulcis* stems revealed differences in the chlorophyll fluorescence and quenching parameters between the two species. (d) Gas exchange analysis showed that guard cells of *P. arabica* stems tightly regulate water loss under elevated temperatures while maintaining constant and high assimilation rates throughout the stem. Our data show that *P. arabica* uses a distinctive strategy for tree carbon gain *via* stem photosynthetic capability, which is regulated efficiently under harsh environmental conditions, such as elevated temperatures. These findings are highly important and can be used to develop new almond cultivars with agriculturally essential traits.

## Introduction

Almond, *Prunus dulcis* (Mill.) D. A. Webb, is a major fruit tree crop worldwide, well-known for its high nutritional and health values (Hyson et al., 2002; King et al., 2008; Martínez-González et al., 2008; Estruch et al., 2018). During its domestication process, many of its natural traits were lost. Identifying traits that relate to plant resilience to harsh environments, diseases, and pests in wild relatives, and their implementation in breeding programs can assist in future challenges, awaiting this crop, especially given climate change threats (higher temperatures, prolonged heat waves, water shortage, etc.). Almond growers already face harsh climate conditions, including water shortage (droughts) in some parts of the world [California (Castel and Fereres, 1982); Spain (Moldero Romero et al., 2021)] and insufficient chilling hours, which results in impaired blooming and low yields.

*Prunus arabica* (Olivier) Meikle, also known as *Amygdalus arabica Olivier* (Shmida et al., 2022; USDA, ARS, National Plant Germplasm System, 2022), is a rare almond species that belongs to the Prunus genus and the Rosaceae family (Browicz and Zohary, 1996). It is native to the Asia-Temperate zone, including the Zagros Mountains range from south-eastern Turkey and north-western Iran (Shmida et al., 2022; USDA, ARS, National Plant Germplasm System, 2022). In several countries, including Saudi Arabia, Jordan, Lebanon, Syria, and Israel, it is considered an endangered species (Shmida and Cohen, 1989; El-Sheikh et al., 2019; Shmida et al., 2022). Morphologically, when not grafted, it is a bush, rather than a tree, with few small leaves and green stems that do not develop bark (suberized periderm) for several years (Sorkheh et al., 2009; Karatas et al., 2011; Mirzaei et al., 2017; El-Sheikh et al., 2019). Its fruits are small and bitter. *P. arabica* natively grows on marginal poor soils under harsh conditions. On one hand, *P. arabica* is native to arid and semiarid habitats under dry and hot conditions. On the other hand, this wild almond species is found in mountainous regions, on high altitudes ~1,100–2,300 m, under severe and cold winters (Browicz and Zohary, 1996; Khadivi et al., 2022). *P. arabica* resilience to these severe growth conditions has been attributed to various anatomical, physiological, and biochemical characteristics (Sorkheh et al., 2009, 2011; Rajabpoor et al., 2014; Akgun et al., 2018).

One of the most unique and noticeable features of *P. arabica* is its green stems, which stay green (un-barked) all year-round. This unique trait is even more pronounced during the winter and in response to drought conditions after *P. arabica* sheds its leaves (Sorkheh et al., 2009). This behavior is typical in desert plants, which shed their leaves to reduce plant water loss, yet continue to photosynthesize *via* their shoots (Ávila-Lovera et al., 2019). This similarity raised the assumption that *P. arabica* green stems have a photosynthetic capability (Sorkheh et al., 2009). Indeed, in a recent detailed genetic and physiological study, we have provided for the first time experimental evidence supporting *P. arabica* stem photosynthesis capability [SPC, (Brukental et al., 2021)]. Our study showed that *P. arabica* green stems are photosynthetically active all year round, including during the winter months after the tree sheds its leaves and enters its dormancy period. Furthermore, our genetic study showed that *P. arabica* SPC is genetically inherited and can be used in breeding programs.

It is known that CO_2_ fixation in stems positively contributes to the plant's carbon economy. There are two types of CO_2_ fixation processes within stems, which are reflected by anatomical and physiological differences (Ávila et al., 2014). (a) Stem Recycling Photosynthesis (SRP), also termed corticular photosynthesis, which occurs in a wide variety of plants (Schaedle, 1975) and involves the reassimilation of CO_2_ from respiration (Nilsen, 1995; Ávila et al., 2014), in suberized, stomata-less stem tissue (Saveyn et al., 2010; Ávila et al., 2014; Ávila-Lovera et al., 2019). (b) Stem Photosynthetic Capability [SPC, Brukental et al. (2021)], also known as Stem Net Photosynthesis (SNP) (Ávila et al., 2014)], which includes gas exchange and assimilation of atmospheric CO_2_ *via* stomatal pores within stem epidermis. On a whole tissue basis, both SRP and SPC contribute to the carbon economy of the plant. Yet, the contribution SRP, which reduces the flux of CO_2_ from woody tissues to the atmosphere by refixation (Sprugel and Benecke, 1991), is minor compared to SPC. Indeed, SRP can recycle 7 to 123% of the respired CO_2_, but its net photosynthetic rate is negligible compared to leaves (Ávila-Lovera et al., 2019). On the other hand, SPC can reach up to 60% of leaves' photosynthetic rates (Ávila et al., 2014), and even more (Brukental et al., 2021).

The contribution of SPC to the whole plant physiology, in particular under harsh environments, was explored in several types of plants. Different pieces of evidence support the contribution of SPC with extra carbon gain to cope with various detrimental environmental conditions. It was shown that SPC of deciduous desert plants provides continuous photosynthetic activity under dry conditions, even when leaves, the main photosynthetic organ, are absent (Nilsen, 1995; Ávila-Lovera et al., 2019). Moreover, it was found that the relative carbon gain *via* SPC increases in the deciduous shrub *Spartium junceum* in response to water-deficit conditions (Nilsen and Bao, 1990). The role of SPC as a major source of carbon gain was found to be crucial in certain species. For example, with the beginning of the dry season, the native Californian desert shrub, *Justicia california*, loses its leaves and relies on its green stems as the sole photosynthetic tissue for 7 months (Tinoco-Ojanguren, 2008). Interestingly, the generation of extra carbon gain by stem photosynthesis, in particular when leaves are absent, plays a substantial role in bud development, flowering, root development, as well as recovery after herbivory (Bossard and Rejmanek, 1992; Tinoco-Ojanguren, 2008; Saveyn et al., 2010; Kocurek et al., 2020). It is most likely that SPC evolved to support plant growth and survival in certain harsh habitats. Zheng et al. (2021) further supported this physiological advantage of SPC by showing that *M. micrantha* uses its high-stem gross photosynthetic levels, following defoliation as an ecological advantage, and further advances its high invasion rates.

Leaves are the primary plant tissue for transpiration and carbon fixation in most plant species. In most deciduous trees, like almonds, carbon is fixed by the leaves during the summer and is stored for the winter, mainly as starch in tree stems and roots. By the end of the winter, trees drop their leaves and rely on their residual carbohydrate resources to fuel bud-break, bloom, and the early stages of vegetative growth (Ito et al., 2012; Tixier et al., 2017, 2020; Fernandez et al., 2018; Amico Roxas et al., 2021). This fuel reservoir is the only energy source for the deciduous tree until new leaves emerge and become fully functional.

With climate change, the increase in world temperature (IPCC, 2021), water shortage, and increase in dry-land areas, bring severe challenges to almond growers (Goldhamer and Fereres, 2017). Water shortage was found to induce leaf drop in commercial almond species in the summer (Castel and Fereres, 1982; Moldero Romero et al., 2021). Furthermore, warm winters enhance tree respiration and impair productivity (Luedeling et al., 2009; Benmoussa et al., 2018). With no photosynthetic organs (leaves) during dormancy, to regain carbon loss, these conditions challenge the almond tree's carbon reserves, and, eventually, its productivity (Zwieniecki and Secchi, 2015; Sperling et al., 2019). Photosynthetic stems, which have never been described before in deciduous fruit crop trees (until our work by Brukental et al., 2021), can thus contribute to the net carbon gain, and increase the tree resilience to extreme environmental conditions.

In this study, we carried out a detailed comparison between two almond species, the commercial cultivar “Um-el-Fahem” (*P. dulcis*) and the rare wild *P. arabica*, focusing on its unique SPC trait. Here, we bring physiological and anatomical evidence that these two almond species use two distinctive strategies for carbon gain. The commercial almond, *P. dulcis*, like other deciduous fruit trees, is dependent on its leaves for CO_2_ assimilation. On the other hand, the wild almond, *P. arabica*, uses both its leaves and its stems for efficient carbon gain. Its stems assimilate remarkable levels of CO_2_ *via* Stem Photosynthetic Capability (SPC) (Brukental et al., 2021), which involves the uptake of atmospheric CO_2_ *via* stomata pores within stem epidermis.

## Materials and methods

### Plant material

Two almond species were used in this study. (1) *Prunus arabica* (Olivier) is an endangered species, which grows in Israel in the Judean desert. In July 2014, stem cuttings were taken from one specimen. The budwood was then top grafted on GF.677 rootstocks and planted in the almond orchard at Newe Ya'ar Research Center, Israel. (2) The commercial cultivar *Prunusdulcis* (Mill.) D. A. Webb ‘Um-el-Fahem’ (grafted on GF.677 rootstock) was used as a reference in all the experiments. The experiments were conducted in two almond plots in Newe-Ya'ar. In a mature 5-year-old plot, and in a parallel experimental setting, in a young 3-year-old orchard (planted on 2017). Each of the two plots includes 2 trees from each cultivar (*P. dulcis* and *P. arabica*). In each set of experiments, multiple stems and leaves were measured (as described in each figure legend) from two trees of each species. Newe-Ya'ar is located in the western Yizre'el Valley at the elevation of 100 m above the sea level, characterized by a Mediterranean, subtropical climate. Orchard trees are grown in clay grumusol (vertisol) soil. During this research, the minimum and maximum temperatures were 6°C (43°F) during January and 33°C (91°F) during August. Mean annual precipitation from November to March was 580 mm, while, during the dry season (April to October), the orchard was fertigated and irrigated according to standard procedures (an average of 69 m^3^/dunam for the whole season).

### Surface area evaluation of the aboveground tree parts

The surface area of leaves, green stems, and barked stems was evaluated in mature *P. dulcis* and *P. arabica* trees at the Newe-Ya'ar almond collection during the beginning of July 2019, when the trees were fully covered with leaves. Surface area measurements were conducted on a quarter of the tree (33 *P. arabica stems* and 9 *P. dulcis* stems), and the results were then multiplied by four. All leaves were collected and weighed. To evaluate the total surface area of tree leaves, 50 random leaves were sampled, weighed, and measured for their area, using a computer scanner and the “Tomato-Analyzer” software program version 4 (Brewer et al., 2006). Based on the computed weight of all tree leaves, the computed weight of a single leaf, and the computed surface area of a single leaf, the total surface area of the leaves was evaluated for *P. dulcis* and *P. arabica* trees. To estimate the total surface area of green stems and barked stems in both species, the length and the perimeter of green/barked stems were measured in the field. Next, the surface area of green/barked stem was calculated according to the formula of a cylinder surface area (*A* = 2π*rh*; *r* = radius, *h* = height). The results of all internodes were averaged, and the tree total green/barked stem area was computed.

### Stomatal imprints

Stomatal density measurements were made on a subset of leaves and stems using a rapid imprinting technique (Geisler and Sack, 2002), which allowed the reliable scoring of hundreds of stomata at the same time. In brief, light-bodied vinyl polysiloxane dental resin (Heraeus-Kulzer, https://www.kulzerus.com) was attached around the stems and on leaf abaxial and adaxial surfaces. After drying, the resin imprints were covered with transparent nail polish, which was removed once dried. The nail-polish stomatal imprints were put on microscope slides and photographed under a bright-field Olympus BX61 microscope (https://www.olympus-ims.com) with an Olympus DP73 color camera (https://www.olympus-lifescience.com). The area was calculated based on a micrometer slide. Stomatal images were analyzed using IMAGE J (http://rsb.info.nih.gov/ij).

### Scanning electron microscopy

For SEM analysis, stem samples fixed in formalin-acetic-alcohol (FAA) (Bello et al., 2017) were passed through a series of ethanol solutions (50, 70, 90, 95, and 100%). The dehydrated samples were then dried in a quorum K850 critical point dryer (https://www.quorumtech.com) using liquid CO_2_. The fully dried samples were mounted on aluminum stubs with double-sided adhesive tapes and then coated with gold using a quorum SC7620 sputter coater (https://www.quorumtech.com) supplied with argon gas. Photomicrographs of the samples were prepared using a Hitachi TM3000 SEM tabletop microscope (https://www.hitachi-hightech.com).

### Chlorophyll content measurement

Determination of the chlorophyll (chl) level in *P. dulcis* and *P. arabica* stems and leaves was done as described in Porra (2002) with the following modifications. Leaves were sampled, scanned, and immediately frozen in liquid nitrogen. Approximately, 3 cm (in length) stem-cuts were collected from the 1^*st*^-year (current year growth), and the 2^*nd*^ year stems (stems from last year growth). Each sample was weighed, measured for its diameter using a caliber, and immediately frozen in liquid nitrogen to arrest any metabolic activity. The surface area of both leaves and stems was calculated as described above (in surface area evaluation). Leaves and stems were then grounded in liquid nitrogen, using a mortar and pestle (leaves) or Ika A11 basic Analytical mill (IKA®-Werke GmbH & Co. KG, Germany; stems). Chl was extracted in 80% acetone in phosphate buffer pH 7.8, at 4°C in the dark. The supernatant from two consecutive overnight extractions was combined for each sample. Absorbance (A663.6/A646.6) was measured (CARY 50 Bio UV-visible Spectrophotometer, Agilent Technologies Inc., Santa Clara, CA, USA), and chl concentration was calculated (Porra, 2002).

### Chlorophyll autofluorescence confocal microscope analysis

Fresh cross-sections of 15 μm were made from 1^*st*^ and 2^*nd*^-year stems using a sliding microtome (Reichert Wien, Shandon, Scientific Company, London). Cross-sections were then mounted on slides in water and imaged under a confocal microscope. Images were acquired using an IX81 fully automated Olympus microscope equipped with a 488-nm argon laser, using a BA 660 IF emission filter for chl red autofluorescence, and a UPlanApo10X (NA0.4) objective. Transmitted light images were acquired using Nomarski differential interference contrast. An image analysis was carried out using the flow view software (Olympus).

### Histology

For anatomical analyses, *P. arabica* and *P. dulcis* 1^*st*^ and 2^*nd*^-year stems were cut to ~1-cm length fragments and fixed for 2 days in FAA (10% formaldehyde, 5% acetic acid, 50% ethanol, v/v in water) under 4°C in the dark. Fixation was followed by incubation in an increasing concentration of ethanol (50, 70, 90, 95, 100, and 100% × 2) and then by the exchange of ethanol with Histoclear (xylem substitute). The samples were then embedded in paraffin, and 12-μm cross-sections were prepared using a Leica RM2245 microtome (Leica Biosystems, Nussloch, Germany). Sections were stained with safranin and fast green (Ruzin, 1999), and photographed under a light microscope (Leica-DM500, Heerbrugg, Switzerland), with an ICC50 HD camera at various magnifications (10 ×, 20 ×, and 40 ×).

### Pulse-amplitude-modulated chlorophyll *a* fluorescence

Stems at different developmental stages (1^*st*^, 2^*nd*^, or 3^*rd*^ year) and leaves from *P. arabica* and *P. dulcis* were collected from the orchard and kept hydrated by immersing their cut ends\petioles in water. A ~5-cm piece of each stem replicate (*n* = 3–4) or whole leaves (*n* = 3–4) were used for the chl fluorescence measurements using a Maxi Imaging PAM chl fluorometer (Heinz Walz GmbH, Effeltrich, Germany, https://www.walz.com/). The stems and leaves were dark-adapted for 20 min and then subjected to an induction curve analysis followed by a dark recovery period. The minimal fluorescence signal in the dark (F0) and the maximal fluorescence signal (Fm) were recorded before and after a saturating pulse of 6,000 μmol photons m^−2^ s^−1^ (600-ms duration), respectively. An actinic light of 530-μmol photons m^−2^ s^−1^ was turned on after 40 s of dark incubation, following the saturating pulse, generating a steady-state fluorescence signal (Ft). Subsequent maximal chl fluorescence (Fm') was recorded at 20-s intervals by applying saturating pulses on top of the actinic light. The maximum quantum yield of PSII (Fv/Fm) and non-photochemical quenching of fluorescence (NPQ) were calculated according to Maxwell and Johnson (2000), as Fv/Fm = (Fm – F0)/ Fm and NPQ = (Fm – Fm′)/Fm′.

### Gas exchange measurements

Gas exchange measurements were conducted on *P. arabica* and *P. dulcis* intact (attached to the tree) stems and leaves in the almond orchard. In all experiments, *P. arabica* and *P. dulcis* were grown side by side, under the same growth conditions, and analyzed in the same period of time. For stem gas exchange analysis, the leaves were removed 2 days prior to the measurements to eliminate any wounding stress effect.

The response of CO_2_ assimilation and transpiration water loss to temperature increases in *P. arabica* and *P. dulcis* almond leaves and 1^*st*^-/2^*nd*^-year stems was done in the orchard during 2019 early and mid-summer (between 9:00 a.m. and 3:00 p.m.), while leaves were fully functional. Using the CIRAS-3, gas exchange systems (PP Systems, Amesbury, MA, USA), measurements were carried out with a PLC3 leaf cuvette (a 1.75 cm^2^ chamber). The following conditions were held constant in the cuvette: photon flux density of 1,200 μmol m^−2^s^−1^ (90% red, 10% blue supplied by the CFM 3 Chl Fluorescence module) and VPD (air vapor pressure deficit) of 3 kPa. Gas exchange measurements were recorded under constant VPD (3 kPa) at varying temperatures set in the PP system photosynthetic gas exchange analyzer chamber. For each analyzed stem or leaf, the temperature in the chamber was first set to 28°C, and then increased to 35°C, and, finally, 40°C. Data were logged after stabilization on steady state for each temperature measured (after ~5 min).

Gross photosynthesis of *P. arabica* and *P. dulcis* 1^*st*^-year stems was calculated from gas exchange measurements that were done during February and March 2020. The measurements were carried out with the LI-6800 Portable Photosynthesis System (LI-COR Environmental Lincoln, Nebraska, USA), using the 6-x-6-cm Large Leaf and Needle Chamber equipped with Licor's Large Light Source. This measurement setting is suitable for thin stems of a 2.5–4.5 mm diameter and provides a good light and gas seal. CO_2_ flux was measured under light and dark conditions to evaluate stem gross photosynthesis, as described in Cernusak and Marshall (2000). The following conditions were held constant in the chamber: relative humidity of 40% and temperature of 16 or 21°C during February or March, respectively (based on the average temperature conditions of each month). First, CO_2_ flux was measured in light with a photon flux density of 1,200 μmol m^−2^ s^−1^ (90% red, 10% blue). Next, the Li-6800 light source was turned off for ~2 min (until stabilization of CO_2_), and data were recorded (referred to dark respiration). Gross photosynthesis was calculated by subtracting the respiration rates from the net photosynthesis rates, as described in Cernusak and Marshall (2000). All measurements mentioned above were done on four stems from each species on 2 separate days (*n* = 8) between 09:30 h and 11:00 h.

Stem *A/Ci* curves (net CO_2_ assimilation as a function of internal CO_2_ concentration) of *P. arabica* and *P. dulcis* 1^*st*^-year stems were measured with the LI-6800 6 x 6 cm^2^ Large Leaf and Needle Chamber equipped with Licor's Large Light Source during April 2021. The stems were harvested, and their cut ends were immediately immersed in water and transferred to the lab for analysis. During gas exchange measurements, the following parameters were held constant: chamber relative humidity of 55%, air temperature of 21°C, photon flux density of 1,200 μmol m^−2^ s^−1^ (90% red, 10% blue). After stabilization under the ambient CO_2_ level (400 ppm), CO_2_ concentration in the chamber was changed in the following sequence: 400, 300, 200, 50, 400, 500, 600, 700, 800, 900, 1,000, 1,300, and 1,600 (ppm). Stabilization time for each CO_2_ level was 2 min. IRGAS (infrared gas analyzers) were matched before each logging. For calculation of the internal CO_2_ concentration (Ci), boundary layer (BL) conductance surrounding the stem segment was calculated as described in Kollist et al. (2007), with the following modifications. A wet filter paper was rolled on a representative stem segment and inserted into the leaf chamber. The transpiration rate from this wet paper was used by the machine to calculate whole leaf conductance to water vapor. This value was then entered into the Li-6800 constants as stem BL conductance. Data obtained were normalized for the stem-projected area.

### Statistics

Statistical analyses, including calculations of means, SDs, the Student's *T-*test, one-way ANOVA, two-way ANOVA, and separation of means by the Tukey–Kramer HSD test were conducted using the JMP^®^ pro 14.0 software (SAS Institute Inc., Cary, NC). The relevant tests for each analysis are described in each figure legend.

## Results

### Morphological comparison between the wild almond *P. arabica* and the commercial cultivar *P. dulcis* suggests distinctive strategies for carbon gain in these two almond species

**Figure 1:**
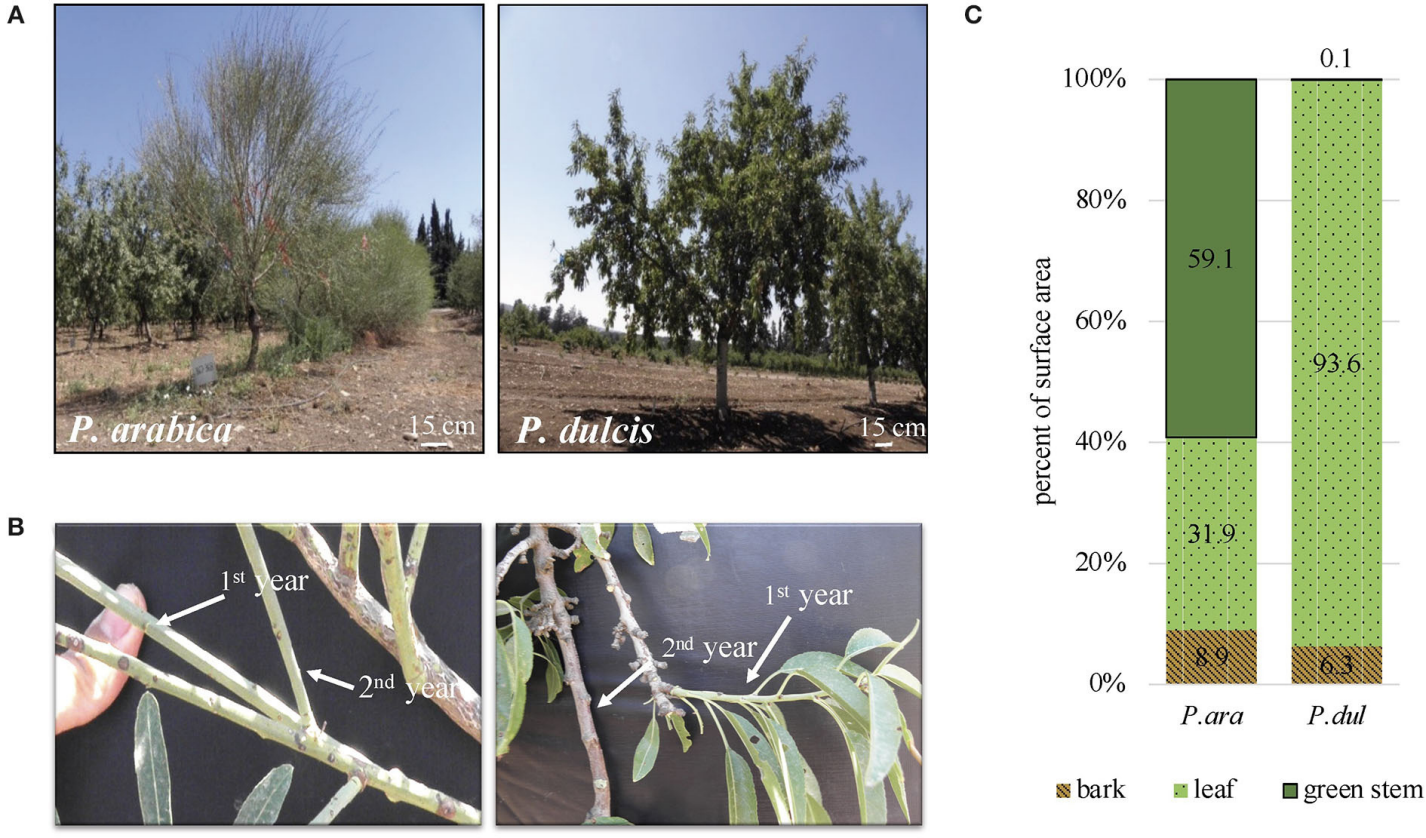
The wild *P. arabica* and the commercial cultivar *P. dulcis* trees show distinctive architecture. Representative pictures of *P. arabica* (*P. ara*) and *P. dulcis* (*P. dul*) **(A)** whole trees and **(B)** 1^*st*^ and 2^*nd*^-year stems **(C)** Surface area measurements of the tree green stems, barked stems, and leaves in *P. arabica* and *P. dulcis*. Results are presented as percent of the total surface area of the aboveground parts of the tree.

In this study, we made a detailed morphological and physiological comparison between two almond species, the wild *P. arabica* and the commercial cultivar ‘Um-el-Fahem’ (*P. dulcis*). On the whole-tree level, *P. arabica* shows a bushy growth structure in nature (Brukental et al., 2021). In our orchard, *P. arabica* and *P. dulcis* were grafted on a GF.677 rootstock and trained on one trunk. Representative pictures of *P. arabica (P. ara*) and *P. dulcis (P. dul)* show the distinctive tree architecture of the *P. arabica* wild almond species (Figures 1A,B). While a typical *P. dulcis* tree is covered with many elongated bright green leaves, the *P. arabica* tree has fewer leaves that are restricted to current year growth. Its leaves are rigid and grayish in color and found to be significantly smaller; ~20% of the surface area of *P. dulci*s leaves (data not shown). It is important to note that the wild *P. arabica* tends to drop its leaves during the dry season. In our fertigated orchard, the leaves remained vital and intact until November. One of the most distinctive features of the wild *P. arabica* is its green stems that stay green all year round. Morphological comparison between the two species reveals that *P. dulcis* young stems are green during their first summer (i.e., 1^*st*^-year stems) and develop a brown corked bark layer by their first winter (i.e., 2^*nd*^-year stems), whereas *P. arabica* 1^*st*^ and 2^*nd*^-year stems remain green (Figure 1B). Stems' shape is distinctly different between the two almond species. While both *P. arabica* and *P. dulcis* 1^*st*^-year stems are thin and flexible, *P. dulcis* stems develop short internodes and become lignified and hardened by their 2^*nd*^ year. *P. arabica* stems are long, thin, and remain relatively more flexible, showing some resemblance in shape to the Spanish broom bush (*Spartium junceum*). To quantify this trait, surface area evaluation of the aboveground tree parts was made in both almond species during the beginning of July 2019, while the trees were fully covered with leaves. The results indicate significant differences in the relative surface area of the leaves, barked stems, and the green stems in *P. dulcis* (~94, 6, and 0.1%) and, in *P. arabica* (~32, 7, and 60%) (Figure 1C). In *P. dulcis*, the leaves occupy 94% of the tree's total surface area. On the other hand, in *P. arabica*, the leaves occupy only 32%, while green stems constitute the major part, with 60% of the tree's surface area.

### *P. arabica* stems possess unique anatomical features that support their photosynthetic abilities

(a) *P. arabica stems are covered with high density of sunken stomata, in contrast to the stomata on P. dulcis stems, which disappear under a thick bark layer by their 2*^*nd*^ *year of development*.

**Figure 2:**
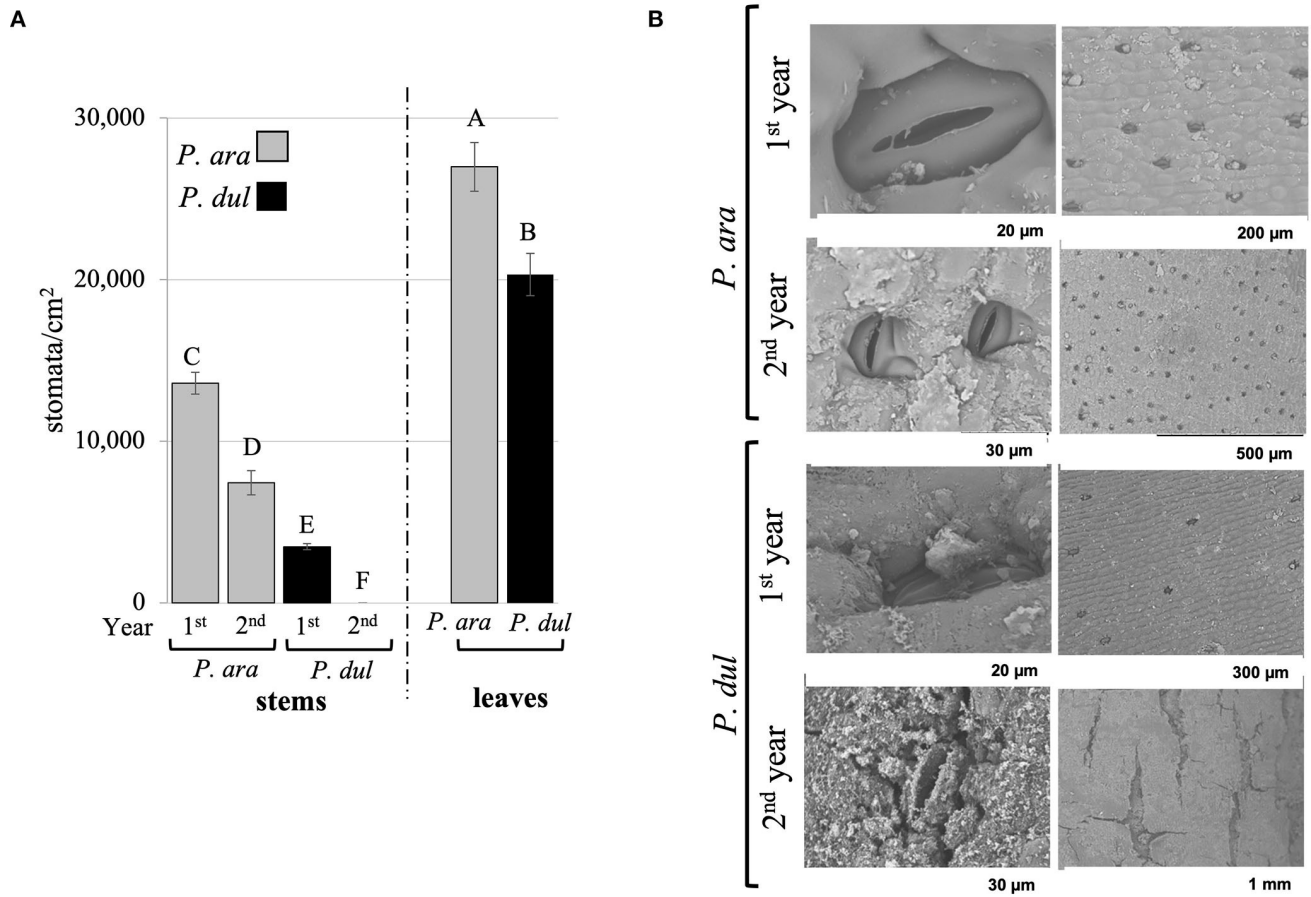
Characterization of the stomata in *P. arabica* and *P. dulcis* stems and leaves. **(A)** Stomata quantification on *P. arabica* and *P. dulcis* stems and leaves. The number of stomata was measured in stems of the 1^*st*^ year (1^*st*^ year) and the second (2^*nd*^ year) and in leaves. Figure presents the mean ± SE of *n* = 30 1^*st*^-year stems, *n* = 8 2^*nd*^-year stems, and *n* = 11 leaves, in each almond species. Capital letters above columns represent statistically significant differences (*p* < 0.05 *t-test*). **(B)** Scanning electron microscope analysis of *P. arabica* and *P. dulcis* 1^*st*^ and 2^*nd*^-year stems indicates sunken stomata in the green 1^*st*^-year stems in both species. In addition, although stomata remained on adult stems of *P. arabica*, even in their 2^*nd*^ year, *P. dulcis* stomata were covered with cork. Representative images are shown.

Our previous study showed that *P. arabica* assimilates CO_2_ through its green stems all year round (annual average of 8 ± 0.19 μmol CO_2_ m^−2^ s^−1^), compared to negligible levels in stems of *P. dulcis* (annual average of 0.5 ± 0.05 μmol CO_2_ m^−2^ s^−1^) (Brukental et al., 2021). To further investigate the physiological basis for these differences, *P. arabica* and *P. dulcis* stems and leaves were analyzed for their stomatal density using a rapid imprinting technique (Geisler and Sack, 2002). Results revealed a significantly greater stomatal density in *P. arabica* stems compared to *P. dulcis. P. arabica* 1^*st*^-year stems had 4 times the stomatal density of *P. dulcis* (13,600 and 3,400 stomata/cm^2^, respectively). Interestingly, while *P. arabica* 2^*nd*^-year stems contained high density of stomata (7,440 stomata/cm^2^), a negligible number of stomata were detected on *P. dulcis* 2^*nd*^-year stems (Figure 2A). Stomatal imprint analyses of *P. arabica* and *P. dulcis* leaves revealed that, in both species, the stomata are concentrated on the abaxial side (Figure 2A), while no stomata were detected on the adaxial side of the leaves (data not shown). However, differences in leaf stomatal density were observed between the two species, with a significantly higher level by 1.3-fold in *P. arabica* compared to *P. dulcis* (27,000 and 20,300 stomata/cm^2^, respectively; Figure 2B). Nevertheless, their stomatal size was similar, with an average length of 22 and 23 μm for *P. arabica* and *P. dulcis*, respectively (data not shown).

Scanning-electron-microscopy (SEM) revealed that the stomata in *P. arabica* and *P. dulcis* stems are sunken within the cuticular layer (Figure 2B). In agreement with stomatal imprint results (Figure 2A), *P. arabica* stems show a significantly greater stomatal density than *P. dulcis* (Figure 2B). Furthermore, the identified stomata on 1^*st*^-year stems of *P. dulcis* were not present on their 2^*nd*^ year due to secondary growth and formation of the periderm, whereas stomata on 2^*nd*^-year stems of *P. arabica* remained morphologically intact (Figure 2B). Interestingly, analysis of stomatal length in SEM images of *P. arabica* and *P. dulcis* 1^*st*^-year stems revealed no significant differences (average of 26 μm; data not shown).

(b) *Unlike P. dulcis stems, P. arabica stems contain significantly higher levels of chlorophyll compartmentalized to a mesophyll-like, chloroplast-rich parenchyma layer*.

**Figure 3:**
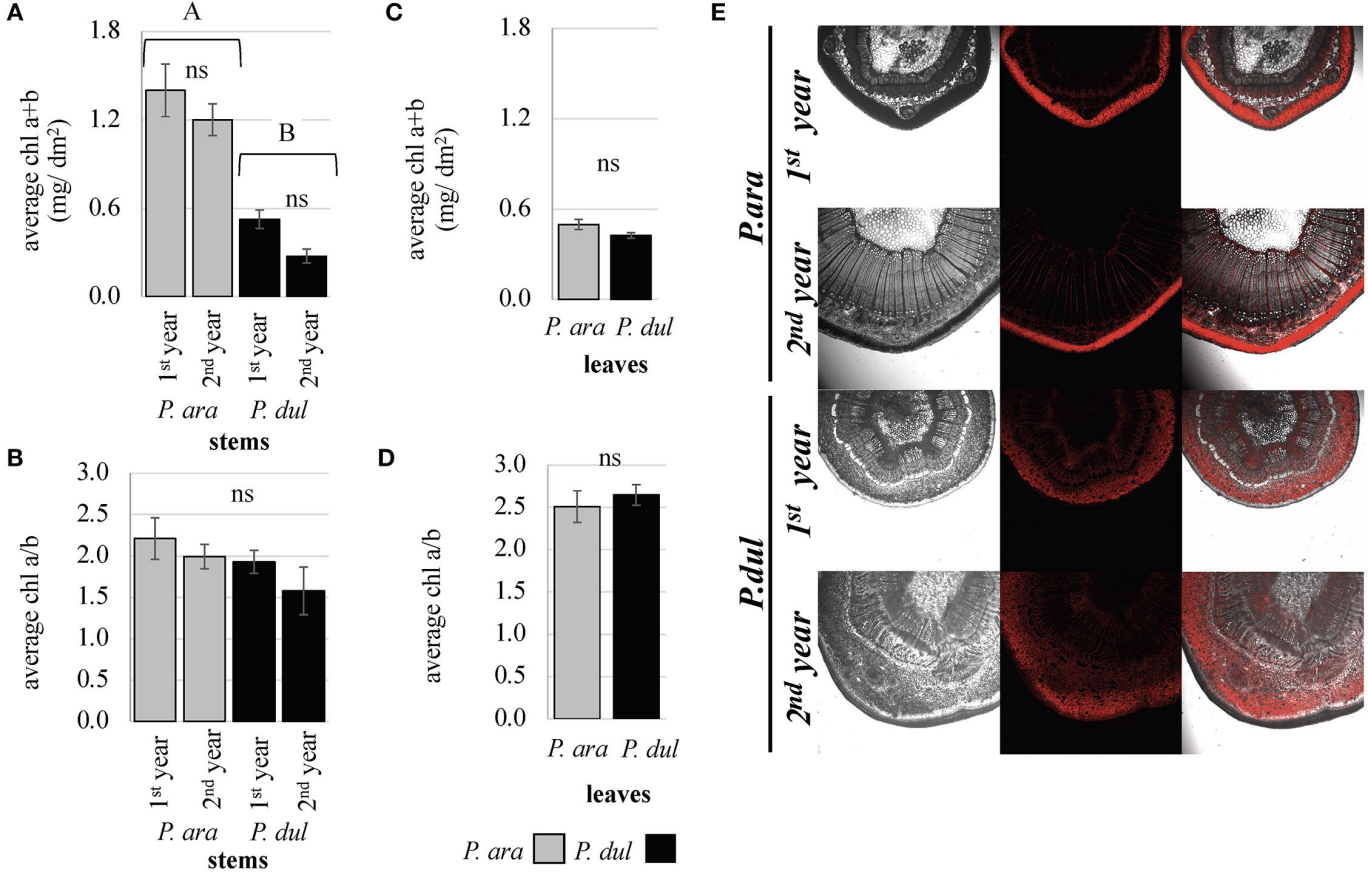
Chlorophyll levels and distribution in *P. arabica* stems and leaves compared to *P. dulcis*. Chlorophyll a and b levels of *P. arabica* and *P. dulcis* 1^*st*^ and 2^*nd*^-year stems **(A)** and fully developed leaves **(C)**. The chlorophyll a/b ratio of *P. arabica* and *P. dulcis* 1^*st*^ and 2^*nd*^-year stems **(B)** and fully developed leaves **(D)**. Data are the means ± SE of 4 samples of leaves/stems from each species. Each sample consisted of 1 leaf or stem, except for the *P. arabica* leaf sample, which consisted of 4 leaves. Statistical analysis of the data was carried out using one-way ANOVA (leaves) and two-way ANOVA (stems). Different letters above the bars indicate significant differences (Student's *T-*test, *p* < 0.05). ns, non-significant. No interaction between almond species and stem age was found in 2-way ANOVA. **(E)** Confocal microscopy reveals a distinguished chlorophyll (red-autofluorescence) distribution in *P. arabica* stems, which is concentrated under the epidermis in the parenchyma layer, while, in *P. dulcis*, the chlorophyll was distributed among different stem layers.

For intact stem photosynthesis, once the stomata are functional and gas exchange occurs, further reactions depend on the functionality of the chloroplasts, with a good proxy being the amount of chl and its distribution in the stem tissues. Our data show that the chl level is significantly higher in the stems of *P. arabica* relative to *P. dulcis* stems (Figure 3A). In *P. arabica* 1^*st*^ and 2^*nd*^-year stems, the chl level (chla + b) per area unit reached 1.4 and 1.2 mg/dm^2^, respectively. On the other hand, in *P. dulcis* 1^*st*^- and 2^*nd*^-year stems, chl content was much lower −0.5 and 0.3 mg/dm^2^, respectively. In *P. arabica* stems, the chl a/b ratio was 2.21 for 1^*st*^-year stems and 1.99 for 2^*nd*^-year stems (Figure 3B). In stems of *P. dulcis*, the chl a/b ratio was somewhat lower (although not statistically significant), 1.93 and 1.58 for 1^*st*^ and 2^*nd*^-year stems, respectively. In leaves, chl levels per area and the chl a/b ratio were found to be similar in both species. The leaf chl level was 0.5 mg/dm^2^ in *P. arabica* and.4 mg/dm^2^ in *P. dulcis* (Figure 3C), with an chl a/b ratio of 2.5 and 2.65, respectively (Figure 3D). The chl a/b ratio was significantly higher in *P. dulcis* leaves (2.65) compared to its stems (1.93 and 1.58 for 1^*st*^ and 2^*nd*^-year stems, respectively). On the other hand, no statistical difference was observed between the chl a/b ratio of *P. arabica* leaves (2.5) and stems (2.21 for 1^*st*^-year stems and 1.99 for 2^*nd*^-year stems).

To examine the distribution of the chl pigment within the different stem tissues, fresh microtome cross-sections from *P. arabica* and *P. dulcis* stems were examined under confocal microscopy. Our investigation revealed a different distribution of chl autofluorescence within *P. arabica* and *P. dulcis* stems. While, in *P. arabica* stems, the chl was concentrated predominantly under the epidermis in the parenchyma layer, in *P. dulcis*, the chl was distributed among the different stem layers, starting from the chlorenchyma, through the phloem and xylem rays, and deep into the stem pith (Figure 3E). This experiment was repeated in April 2019, May 2019, and July 2020 and showed similar results.

(c) *P. arabica stems consist of stomata that open to a chloroplast-rich palisade-like parenchyma layer, while P. dulcis stems develop a thick bark layer*.

**Figure 4:**
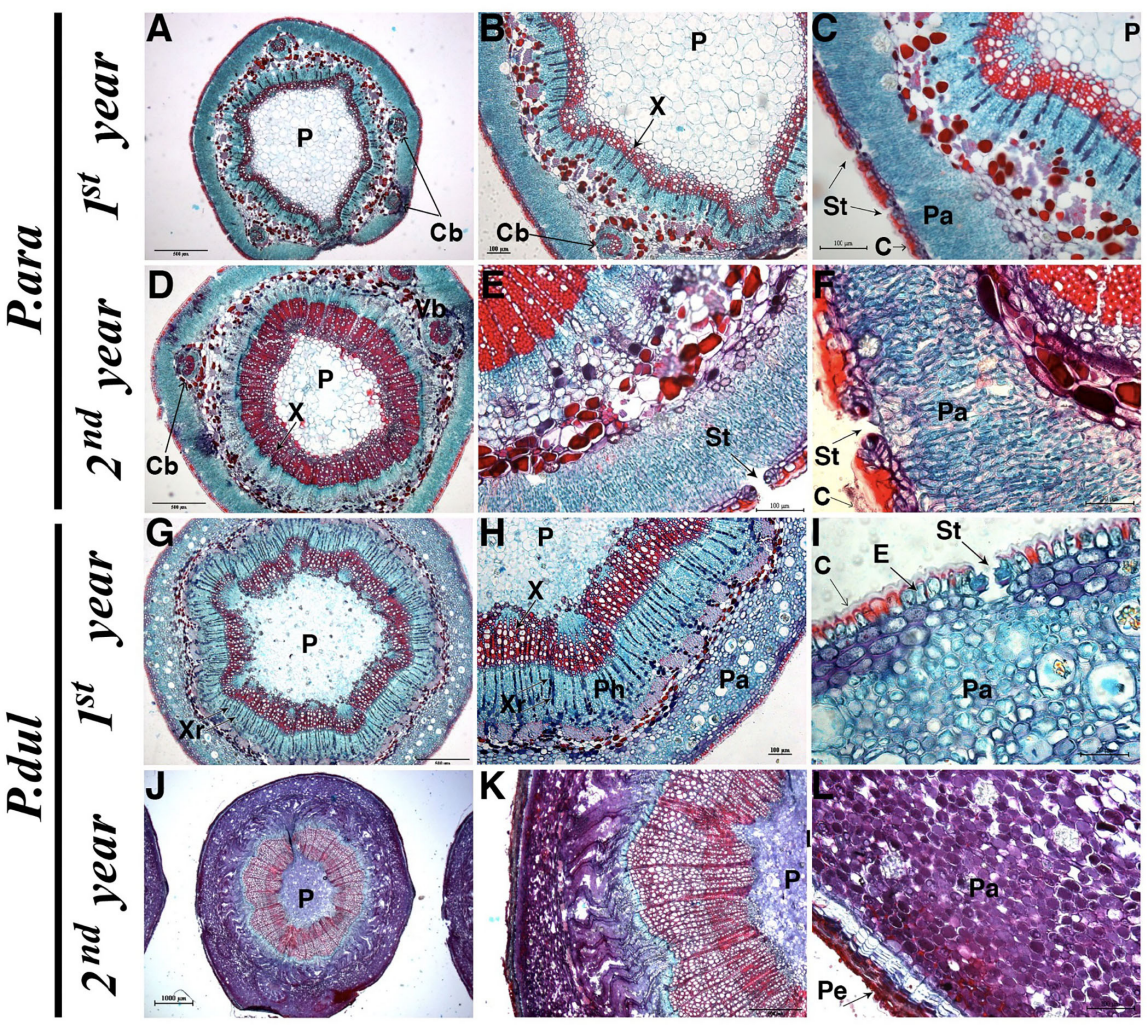
Histological analysis of cross sections in young and mature (1^*st*^ and 2^*nd*^-year) stems of *P. arabica* and *P. dulcis*. Safranin-O/fast green stain. Letters on cuts indicate: C, Cuticula; Cb, Cortical bundles; E, Epidermis; P, Pith; Pa, Parenchyma; Pe, Periderm; Ph, Phloem; St, Stomata; X, Xylem; Xr, Xylem ray. Sizes for scale bars: **(A,D,G,K)** 500 uM; **(B,C,E,H,L)** 100 uM; **(F,I)** 50 uM; **(J)** 1,000 uM.

To further examine stem anatomy, cross-sections of *P. arabica* and *P. dulcis* 1^*st*^ and 2^*nd*^-year stems were stained with safranin-O/fast green and visualized under a light microscope. The analyses revealed distinctive anatomical differences between the two almond species (Figure 4). In the 1^*st*^ and 2^*nd*^-year stems of *P. arabica*, a high number of stomatal pores were visualized within the stem epidermal layer. These pores were opened to a palisade-like parenchyma layer, rich in chloroplasts (light-blue round shapes inside the cells). 1^*st*^-year stems of *P. dulcis* show some similarity in their structure and histological features to the 1^*st*^-year stems of *P. arabica*, having stomatal pores within their stem epidermal layer that is protected by cuticular film. Yet, parenchyma cells of *P. dulcis* 1^*st*^-year stems did not show the “palisade-like” cell structure as in *P. arabica* but were round shape. Furthermore, while no significant histological differences were observed between the 1^*st*^ and 2^*nd*^-year stems of *P. arabica*, 2^*nd*^-year stems of *P. dulcis* showed pronounced developmental changes. These changes include developmental-alternation/maturation of the parenchyma cells (observed by the changes in cell color to purple) and cork formation, which includes the periderm (consisting of three different layers: I. phellem—cork cambium, II. The phellogen meristematic cell layer, and III. phelloderm—known as the secondary cortex). Note that these histological features were observed solely in 2^*nd*^-year stems of *P. dulcis*, but not in *P. arabica*. Another noticeable variation between stems of both species is that *P. arabica* stems contained five additional cortical bundles, while *P. dulcis* contained solely the main vessel, and no cortical bundles were observed (Figures 4A,D).

### *P. arabica* and *P. dulcis* stems exhibit differences in chlorophyll fluorescence and quenching parameters

**Figure 5:**
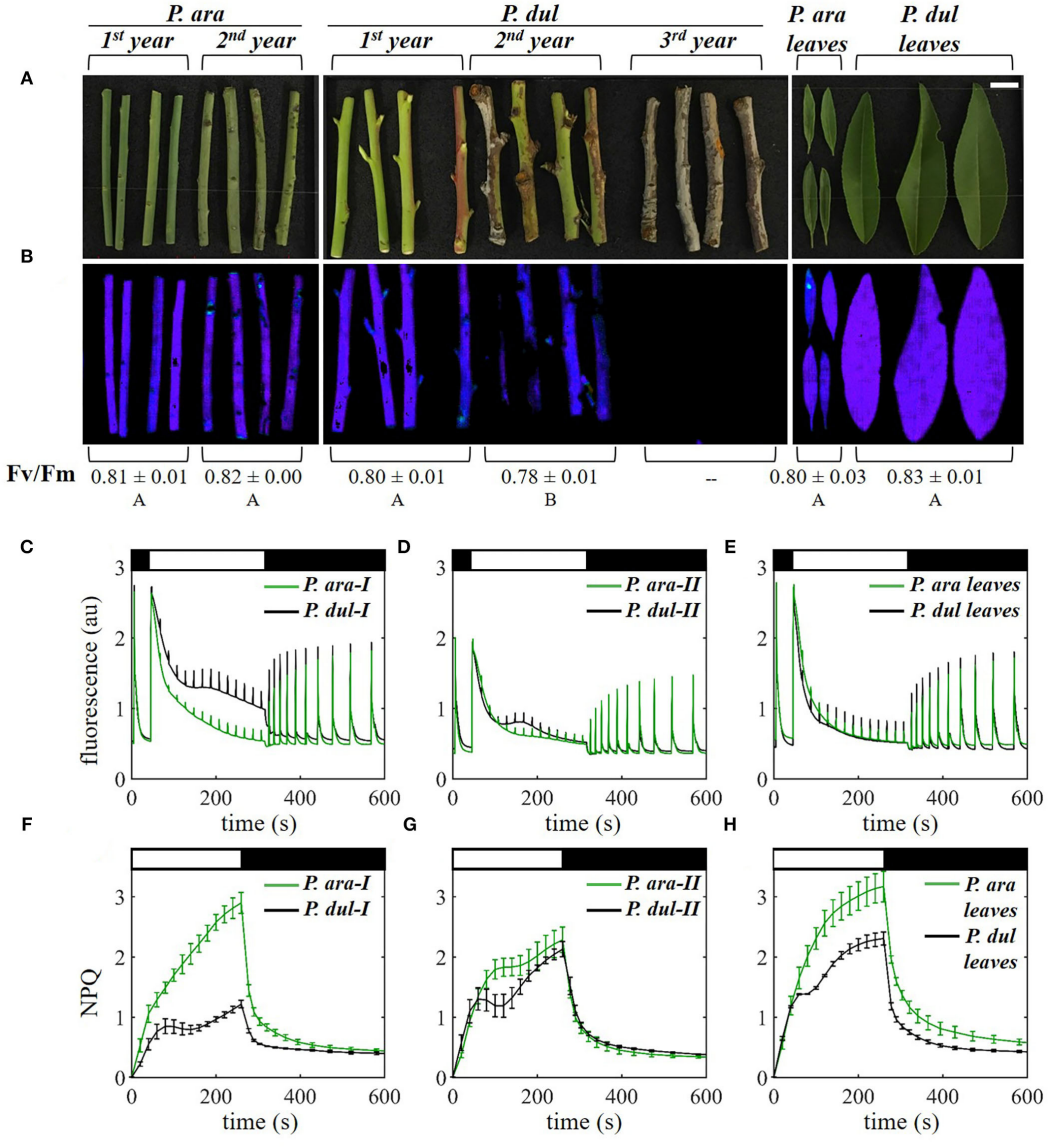
Chlorophyll *a* fluorescence analysis of *P. arabica* and *P. dulcis* stems and leaves. **(A)** Images of 1^*st*^ and 2^*nd*^-year stems of *P. ara*; 1^*st*^, 2^*nd*^, and 3^*rd*^-year stems of *P. dul*; and leaves of the two species in May 2019. A scale bar = 1 cm. **(B)** Corresponding fluorescence images depicting the maximum quantum yield of photosystem II (Fv/Fm) in false color. The mean Fv/Fm values (±SD) of the different stage branches and leaves are denoted below the images; Different letters indicate a statistically significant difference (*p* < 0.05) for stems and leaves separately. **(C–E)** Maximum chlorophyll fluorescence (Fm') induction curve analysis of **(C)** 1^*st*^ (I)-year stems, **(D)** 2^*nd*^ (II)-year stems, and **(E)** leaves, recorded in the morning following dark adaptation. Curves show the mean of 3-4 stems or leaves per group. **(F–H)** Non-photochemical quenching [NPQ, calculated as (Fm-Fm')/Fm'] induction and relaxation of the same stages shown in **(C–E)**. NPQ curves shown are means ± SE (*n* = 3-4 branches or leaves). Black/white bars at the top of the graphs indicate switching on and off the actinic light (a white bar = 530 μmol photons m^−2^s^−1^).

To investigate the photosynthetic performance of the two almond species, chlorophyll fluorescence analysis was performed on the leaves and on the 1^*st*^ and 2^*nd*^-year stems of *P. arabica* and *P. dulcis*, using an imaging PAM (Figure 5). The maximum quantum efficiency of photosystem II (PSII), Fv/Fm, is visually depicted in Figure 5B. Fv/Fm of the stems at the different developmental stages was ~0.8, similar to non-stressed leaves, aside from the 2^*nd*^-year stems of *P. dulcis*, where it was somewhat lower. The value in leaves of both species was also typical to non-stressed leaf tissues (Figure 5B). Leaves of both species exhibit typical fluorescence traces, where the effect of the actinic light (AL) on the photosynthetic system is characterized by an initial fluorescence rise followed by a decline of the fluorescence signal, indicative of the onset of the carbon fixation reactions (“the Kautsky effect”) (Figure 5E). *P. dulcis* leaves exhibit a slightly faster fluorescence decline, possibly reflecting more efficient induction of photochemistry in these leaves. Notably, *P. arabica* 1^*st*^-year stems exhibit fluorescence traces that are very similar to leaves (green traces in Figures 5C,E), suggesting that carbon fixation in *P. arabica* 1^*st*^-year stems is like their leaves. On the other hand, the 1^*st*^-year stems of *P. dulcis* differ from those of *P. arabica* (Figure 5C). In the former, the initial decline in fluorescence following application of the AL is slower and is followed by a further decline in the rate of fluorescence quenching after the 150-s time point. This indicates that fluorescence quenching by photochemical and non-photochemical components is less efficient in 1^*st*^-year stems of *P. dulcis* than those of *P. arabica*. The fluorescence rise after the onset of the AL in the 2^*nd*^-year stems in both species is similar; however, the fluorescence quenching is somewhat lower in *P. dulcis* (Figure 5D). Second-year stems of both species exhibited lower maximum chl fluorescence (Fm) as compared to 1^*st*^-year stems (Figure 5C) or leaves (Figure 5E). The chl content of the stems of each species did not considerably differ between the 1^*st*^ and 2^*nd*^ years (Figure 3A), and, therefore, the reduced Fm values in 2^*nd*^-year stems are likely not due to a change in the level of chl. As fluorescence emission is dependent on the optical properties of the tissue; in this case, stem tissue, the lower Fm, may be due to the different structures and/or water contents of both 2^*nd*^-year stems as compared to 1^*st*^-year stems. As mentioned above, Fv/Fm, which is not dependent on these tissue characteristics, was generally similar between the different developmental stages (Figure 5B).

Examination of non-photochemical quenching (NPQ) induction and relaxation of the leaves of the two species showed that *P. dulcis* leaves exhibit lower NPQ than *P. arabica* (Figure 5H). As the fluorescence quenching is nearly the same in both types of leaves (Figure 5E), this may be due to higher photochemical quenching in the former, i.e., higher CO_2_ assimilation capacity in *P. dulcis* leaves. NPQ of 1^*st*^-year *P. dulcis* stems is considerably lower than *P. arabica* 1^*st*^-year stems (Figure 5F), in line with a less-efficient fluorescence quenching in *P. dulcis* (Figure 5C). In *P. arabica*, NPQ induction in 2^*nd*^-year stems was slower after ~100 s and reached a lower value than its 1^*st*^-year stems (Figures 5F,G). The opposite was true for *P. dulcis*, with a faster initial NPQ induction reaching a higher level in 2^*nd*^-year stems as compared to 1^*st*^-year stems. The higher NPQ in 2^*nd*^-year stems of *P. dulcis* suggests that, at this stage, the stems possess low photochemical quenching.

### Guard cells of *P. arabica* stems tightly regulate water loss under elevated temperatures while maintaining constant and high assimilation rates

**Figure 6:**
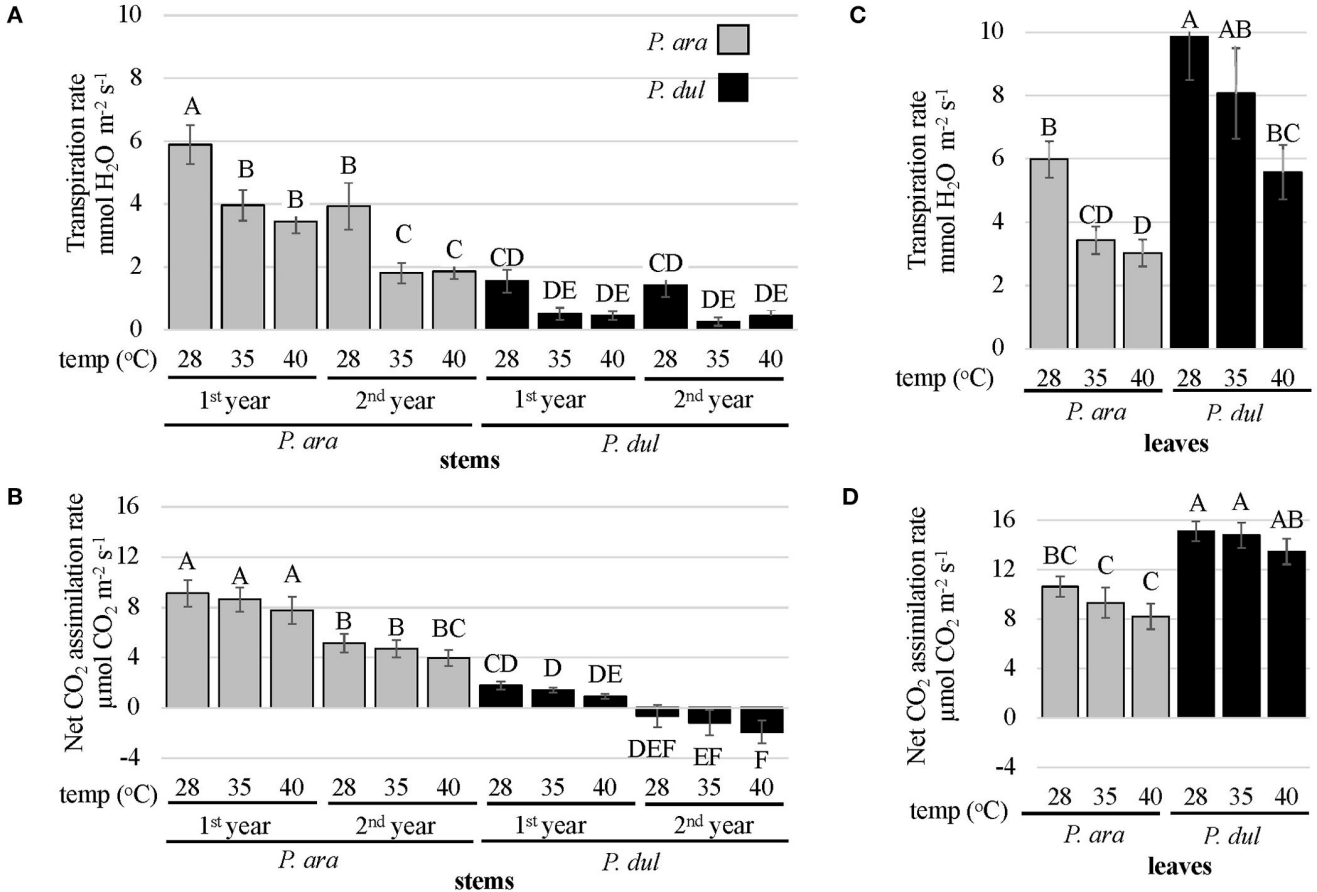
Gas exchange measurements of leaves and stems of *P. arabica* and *P. dulcis*. Gas exchange measurements of leaves and stems of *P. arabica* and *P. dulcis* were performed during the summer season under varied controlled temperatures (28, 35, 40°C). **(A)** The stem transpiration rate (E) full factorial analysis of variance was significant (*R*^2^ = 0.64, *p* < 0.0001). Stem E rates between almond species, stem age, or under different temperatures were highly significant (*p* < 0.0001 for each). The interaction between almond species and stem age was also significant (*p* = 0.0002). **(B)** Stem net CO_2_ assimilation rates (*A*_*n*_) factorial analysis of variance was significant (*R*^2^ =0.67, *p* < 0.0001). Stem *A*_*n*_ levels between the two almond species or between stem age were highly significant (*p* < 0.0001 for each), and did not show significant interaction. **(C)** Leaf transpiration rates (E) factorial ANOVA was significant (*R*^2^ = 0.52, *p* < 0.0001). Leaf E levels between almond species or under different temperatures were highly significant (*p* < 0.0001 for each) and did not show significant interaction. **(D)** Leaves net CO_2_ assimilation rates (*A*_*n*_) factorial ANOVA was significant (*R*^2^ = 0.59, *p* < 0.0001). Leaves A_n_ levels between almond species were highly significant (*p* < 0.0001). Leaves A_n_ levels under different temperatures were significant at *p* = 0.03. Unequal uppercase letters above columns represent statistically significant differences (Tukey HSD performed on all possible combinations, *p* < 0.05). Data show the means ± SE of *n* = 12 branches or *n* = 18 leaves.

We have previously shown, by using gas exchange analysis, that *P. arabica* green stems can act as an active tissue for transpiration and photosynthesis (Brukental et al., 2021). The role of stomatal conductance regulation in leaves and their responses to environmental stimuli has been intensively studied (Zhang et al., 2018; Hsu et al., 2021), and its role in plant fitness and adaptation to its habitat is of great significance. Furthermore, *P. arabica* is adapted to growth in hot desert conditions. To study the physiological responses and contribution of almond stems to harsh environmental conditions, we compared the physiological responses of these two almond species to temperature increments. Gas exchange response measurements were conducted during the summer season on *P. arabica* and *P. dulcis* 1^*st*^ and 2^*nd*^-year stems and their leaves, to temperature increases (28, 35, 40°C) in the gas exchange photosynthetic system chamber, while maintaining constant VPD. The results indicate significant high rates of transpiration and CO_2_ assimilation in *P. arabica* stems compared to *P. dulcis* stems (Figures 6A,B). *P. arabica* stem transpiration rates significantly decreased with temperature increases (Figure 6A). In the 1^*st*^-year stems of *P. arabica*, transpiration rates dropped by ~40% from 5.9 to 4 and 3.5 mmol H_2_O m^−2^ s^−1^ in response to elevated temperatures (28, 35, 40°C, respectively). A similar response was also observed in the 2^*nd*^-year stems of *P. arabica*, showing a reduction in stem transpiration rates only by 51%. Yet, transpiration rates of 2^*nd*^-year *P. arabica* stems were somewhat lower than those detected in 1^*st*^-year *P. arabica* stems, showing 3.9, 1.8, and 1.9 mmol H_2_O m^−2^ s^−1^ at 28, 35, and 40°C, respectively. Transpiration rates of *P. dulcis* stems were extremely low, 2 to 4 times lower than the transpiration rates detected in the 1^*st*^ and 2^*nd*^-year stems of *P. arabica*, respectively (Figure 6A). Interestingly, a relative drop (not significant) in their transpiration rates was noticed in response to temperature increases (*P. dulcis* 1^*st*^-year stems; a drop from 1.5 to 0.5 mmol H_2_O m^−2^ s^−1^ in response to 35 and 40°C; *P. dulcis* 2^*nd*^-year stems; a drop from 1.4 to 0.3 and 0.5 mmol H_2_O m^−2^ s^−1^ in response to 28, 35, and 40°C, respectively).

Net CO_2_ assimilation was found to be significantly higher in both *P. arabica* 1^*st*^ and 2^*nd*^-year stems compared to *P. dulcis*. At 28°C, which is considered an ambient temperature during the summer season in Israel, 1^*st*^ and 2^*nd*^-year stems of *P. arabica* assimilated 9.1 and 5.15 μmol CO_2_ m^−2^ s^−1^, while *P. dulcis* net CO_2_ assimilation was extremely low, and reached 1.8 and −0.6 μmol CO_2_ m^−2^ s^−1^, respectively. Fascinatedly, although the transpiration rates of *P. arabica* stems decreased in response to temperature increases (Figure 6A), net CO_2_ assimilation rates in both *P. arabica* 1^*st*^ and 2^*nd*^-year stems remained high and showed no significant change in response to temperature alterations (Figure 6B). Another interesting point is the significant decrease in the 1^*st*^-year stems net CO_2_ assimilation rates of *P. dulcis* (1.8., 1.4, and 0.9 μmol CO_2_ m^−2^ s^−1^ at 28, 35, and 40°C, respectively) that reached negative values of CO_2_ flux in the 2^*nd*^-year stems (−0.6. −1.2, and −1.9 μmol CO_2_ m^−2^ s^−1^ at 28, 35, and 40°C, respectively). These results suggest that the net CO_2_ assimilation rate in the 2^*nd*^-year stems of *P. dulcis* was lower than the respiratory CO_2_ emitted by the stem.

Results above were obtained from analysis of mature 5-year-old trees (Figure 6). Similar results were obtained from younger 3-year-old trees (Supplementary Figure S1).

When we conducted similar gas exchange measurements in almond leaves, *P. dulcis* showed significantly higher transpiration and net CO_2_ assimilation rates than *P. arabica* under all temperatures analyzed (28, 35, and 40°C). Transpiration was tightly regulated and reduced significantly in high temperatures in leaves of both species (Figure 6C). Assimilation was less affected (Figure 6D). Quantitatively, transpiration in *P. arabica* leaves dropped 2-fold, from 6 mmol H_2_O m^−2^ s^−1^ at 28°C to 3.4 and 3 mmol H_2_O m^−2^ s^−1^ at 35 and 40°C, respectively. In *P. dulcis*, transpiration was 9.9 mmol H_2_O m^−2^ s^−1^ at 28°C and decreased to 8.1 and 5.6 mmol H_2_O m^−2^ s^−1^ at 35 and 40°C (1.8-fold decrease). Slight changes in leaf net CO_2_ assimilation rates were detected in both almond species in response to temperature increases. In *P. arabica* leaves, assimilation rates were 10.6, 9.3, and 8.2 μmol CO_2_ m^−2^ s^−1^ at 28, 35, and 40°C, respectively (decrease, 1.3-fold). In *P. dulcis*, assimilation rates were 15.1, 14.8, and 13.5 μmol CO_2_ m^−2^ s^−1^ at 28, 35, and 40°C, respectively (1.1-fold decrease).

### Net CO_**2**_ assimilation of *P. arabica* stems is dependent on the internal CO_**2**_ concentration

First-year stems of *P. dulcis* possess significantly lower stomatal densities than *P. arabica* stems, which may limit gas exchange. To test whether a limit in CO_2_ gas exchange is the basis for the low CO_2_ assimilation rates in *P. dulcis* stems, gas exchange response measurements were conducted on *P. arabica* and *P. dulcis* 1^*st*^-year stems under different CO_2_ levels [*A/Ci* curve: 400, 300, 200, 50, 400, 500, 600, 700, 800, 900, 1,000, 1,300, and 1,600 (ppm)]. The results of the stem A/Ci curve showed a significant increase in net CO_2_ assimilation rates (A_n_) in *P. arabica* but not *P. dulcis* 1^*st*^-year green stems. To estimate stem photosynthesis capacity, A/Ci data were used to fit the Rubisco maximum carboxylation rate (Vc_max_), the maximum rate of electron transport (*J*_max_), and day respiration (Rd). Results show significantly low Vc_max_, *J*_max_, and Rd levels in *P. dulcis* 1^*st*^-year green stems compared to *P. arabica* (Table 1).

**Table 1:** Photosynthetic parameters of *P. arabica* (*P. ara*) and *P. dulcis* (*P. dul*) stems derived from CO_**2**_ assimilation vs. internal CO_2_ concentration data (A/*C*_*i*_ curves).

**Rd**	**J**_**max**_	**Vc**_**max**_	***n***	**Genotype**
**6.83 ± 1.61**	**251.34 ± 24.89**	**99.99 ± 14.21**	**7**	***P. ara***
**1.16 ± 0.22**	**18.45 ± 2.21**	**5.56 ± 0.78**	**7**	***P. dul***
**0.004**	** <0.0001**	** <0.0001**		***P*** **_value_**

*Vc_max_, maximal carboxylation efficiency, J_max_, maximal electron transport rate, and Rd, respiration in the light. Each value represents mean ± standard errors of seven replication curves for each genotype*.

### Gross photosynthesis of *P. arabica* and *P. dulcis* stems support their function in SPC and SRP-type, respectively

Stem photosynthesis may be attributed to the assimilation of CO_2_ from two different sources: (a) atmospheric CO_2_ by the so-called SPC and (b) internal CO_2_, which is produced by plant respiration termed SRP. To evaluate the ratio between these two processes, gas exchange analyses of 1^*st*^-year stems of *P. arabica* and *P. dulcis* were conducted during the spring (February and March 2020) under light (1,200 μmol m^−2^ s^−1^) and dark conditions. Under light conditions, CO_2_ efflux of *P. arabica* stems was significantly higher than *P. dulcis*, resulting in 7.1 and 0.6 μmol CO_2_ m^−2^ s^−1^, respectively. Under dark conditions, CO_2_ influx of *P. arabica* was significantly higher (more negative) than in *P. dulcis*, with the rate of −1.8 and −0.7 μmol CO_2_ m^−2^ s^−1^, respectively (Supplementary Figure S2). To evaluate stem gross photosynthesis, the absolute values of CO_2_ flux that was measured under the light (i.e., light assimilation) and the dark (i.e., dark respiration) were summed up (as described in Cernusak and Marshall, 2000; Aschan et al., 2001; Damesin, 2003; Ávila-Lovera et al., 2019). The gross photosynthetic rate of *P. arabica* stems reached 8.9 μmol CO_2_ m^−2^ s^−1^ while, in *P. dulcis*, only 1.3 μmol CO_2_ m^−2^ s^−1^.

## Discussion

During the development of agriculture and in the process of breeding for crop improvement, many adaptive traits that were preserved in the wild varieties were lost. Considering climate change with its expected forecasts and in light of the damage that already exists in crop indices, there is an urgent need for the development of new almond varieties. Identification of unique traits in wild tree species that contribute to trees' resilience under harsh environments is of utmost importance and can provide an excellent genetic source for breeding. The wild almond *P. arabica* features various traits that are involved in environmental adaptation. One of the most unique traits is its stem photosynthesis capability (SPC), which we have previously identified and genetically mapped to LG 1 and LG 7 on the genetic map (Brukental et al., 2021). Here, a detailed physiological comparison between the cultivated *P. dulcis* and the wild *P. arabica* reveals different anatomical and physiological characteristics that support their distinctive strategy of carbon gain (i.e., SRP and SPC) described in detail below.

### Morphological comparison between *P. arabica* and *P. dulcis* shows a distinctive tree architecture. While leaves possess the major photosynthetic surface area in *P. dulcis*, green stems possess the greater photosynthetic part in *P. arabica*

The green color of *P. arabica* stems is noticeable all year round and, particularly, during the winter dormancy period (Figure 1 and Brukental et al., 2021). In most woody perennial trees, as in cultivated almonds, the current year's young stems remain green during the summer. However, by the first winter, they change their color and become gray-brown as a result of cork layer formation (i.e., bark). We quantified the proportion between the different aboveground tree parts (i.e., leaves/green-stems/barked stems) in *P. arabica* and *P. dulcis* during the summer. Interestingly, more than 90% of the aboveground surface area is green in both species. However, while, in *P. dulcis*, it is composed mainly of leaves; in *P. arabica*, it is composed of green stems (two-thirds) and leaves (one-third). This ratio suggests that green stems function as the primary carbon-assimilating tissue in *P. arabica*, while, in *P. dulcis*, the leaves are the major photosynthesizing tissue. Interestingly, *P. arabica* leaves were significantly smaller by 4.7-fold than the leaves of *P. dulcis* (data not shown). The feature of small leaves was found in other wild almonds, as in *P. webbi* (average, 4.5 cm^2^, ([Palasciano et al., 2005)] and *P. arabica* close relative, *P. scoparia*, [average length, 2.8 cm; average width, 0.4 cm; (Khadivi-Khub and Anjam, 2014; Zokaee-Khosroshahi et al., 2014)]. This trait was suggested as a plant adaptation to reduce water loss by transpiration in warm and dry habitats and is common in other plant species that use their green stems as their main photosynthetic tissue (Gibson, 1983). The profoundly high proportion of green stems accompanied by few and tiny leaves support the unique physiological strategy of *P. arabica* to invest in the growth of its green stems, its primary carbon source tissue. The contribution of stem photosynthesis in deciduous plants is even greater during the winter, or under stress conditions, when the tree loses its leaves (Nilsen, 1995; Ávila-Lovera and Tezara, 2018).

### *P. arabica* stems carry unique anatomical features that support their photosynthetic capability

Although both SRP and SPC result in CO_2_ assimilation and carbon gain, they differ in their structural and functional traits. Previous studies have shown profound anatomical differences between SRP and SPC (Gibson, 1983; Berveiller et al., 2007; Berry et al., 2021). In a detailed anatomical analysis, Gibson (1983) characterized 30 different plant species, collected from arid and semiarid habitats of North America, that possess perennial green stems for several years. His study concluded that typical SPC species stems have: (a) sunken stomata in a well-developed cuticular epidermal layer (b) a thick chlorenchyma layer and (c) delayed formation of the periderm. Based on our previous results (Brukental et al., 2021), which showed that *P. arabica* green stems assimilate significantly high rates of CO_2_ as compared to *P. dulcis*, we predicted that *P. arabica* stems would possess anatomical features of SPC.

(a) *Stem stomata. SPC requires more than just gas exchange for photosynthesis*.

One of the most distinctive features that account for SPC is functional stomata within the stem epidermal layer. This feature is essential for gas exchange between the stem and the atmosphere for transpiration and CO_2_ assimilation. Our detailed analyses revealed that both almond species develop stomata on their stems. Nevertheless, stomatal density (SD) of the 1^*st*^*-*year stems of *P. arabica* was 4 times higher than in the 1^*st*^*-*year stems of *P. dulcis* (Figures 2A,B). Indeed, high stomatal density in stems is a common feature in SPC-type plant species (Gibson, 1983; Nilsen and Bao, 1990; Yiotis and George, 2011). When we compared between the 2^*nd*^-year stems of *P. arabica* and *P. dulcis*, contrasting developmental phenotypes were found. While high SD was found also on *P. arabica* 2^*nd*^-year stems, the very few stomata that were detected on *P. dulcis* young stems (1^*st*^ year) (Figures 2A,B, 4) were covered under a thick barked layer by the 2^*nd*^ year of stem development. Stem bark is composed of suberins, lignins, and waxes (Ducatez-Boyer and Majourau, 2017), which reduce bark permeability to water and gas (Pereira, 2015; Leite and Pereira, 2017), and, consequently, also, the diffusion *via* stomatal pores. Indeed, the CO_2_ flux rate in the 1^*st*^-year stems of *P. dulcis* was relatively low and even further reduced in their 2^*nd*^ year, resulting in negative values (*A*_*n*_; Figure 6B).

Bark formation on the 2^*nd*^-year stems of *P. dulcis* can explain their negligible transpiration (E) and CO_2_ assimilation (*A*_*n*_) levels. However, these levels were also low in *P. dulcis* 1^*st*^*-*year stems (Figures 6A,B) before any bark was formed (Figure 4I), and gas exchange was available *via* their stomata (Figures 2A, 4I). Although the low stomatal density of *P. dulcis* 1^*st*^*-*year stems (Figures 2A,B) may explain a partial limit in stem gas exchange (compared to *P. arabica* 1^*st*^*-*year stems), it cannot explain the negligible levels of CO_2_ assimilation in *P. dulcis* 1^*st*^*-*year stems (Figure 6B).

Both species show a great number of similarities on their young emerging 1^*st*^-year stems. SEM and histological analysis revealed that both species develop sunken stomata, of the same size, with a deep substomatal chamber (Figures 2B, 4C,I). Sunken stomata are common in plants of dry habitats and have been previously suggested as an adaptation developmental strategy to reduce transpiration and conserve water loss (Jordan et al., 2008). This principle is explained by the higher humidity that sunken stomata may retain, permitting them to open for a longer time under low humidity. With that, opening of stomata for longer periods can contribute to prolonged gas exchange for CO_2_ assimilation, and, as consequence, an improved stem water-use efficiency (Gibson, 1983; Chaves et al., 2016). On the other hand, the feature of sunken stomata may result from the unique epidermal development of the stems in SPC-type species. SPC-type plants are common in high irradiation and high-temperature environments, and their epidermis acquired different characteristics as chemical buildups to reduce the amount of infrared entering the stem. It may be that this developmental feature also results in sunken stomata (Gibson, 1983). Comparison between stomata on *P. arabica* and *P. dulcis* leaves also showed similarity in size and shape. Stomata were located solely on the abaxial side of the leaf in both species. Yet, leaf stomatal densities (SD) were significantly different between the two species, showing higher SD, by 1.3-fold, in *P. arabica* leaves when compared to *P. dulcis* (Figure 2A). Earlier studies revealed significant differences in leaf stomatal frequency and size between wild and cultivated almonds (Palasciano et al., 2005), and even between cultivars of the same species (Oliveira et al., 2018). In this study, the higher SD in *P. arabica* leaves did not result in higher transpiration rates (Figure 6C). These results suggest that additional components, other than SD, affect leaf transpiration rates, and may be attributed to differences in plant hydraulic components (i.e., vascular/extravascular pathways) (Prado and Maurel, 2013). It is important to mention that all our analyses were done on irrigated and fertigated almond trees, and it may be that stomata size and SD will show different characteristics under dry habitat (Casson and Hetherington, 2010; Driesen et al., 2020).

(b) *Chlorophyll levels and distribution in P. arabica* stems—*a functional customization*.

Chl is present at high levels in tree stems (Pfanz et al., 2002; Berveiller et al., 2007; Ávila et al., 2014), and its role in both types of stem photosynthesis (i.e., SPC and SRP) is well-documented (Berry et al., 2021). Nevertheless, the level and the distribution of chl within the different compartments of the stem are distinctive in SPC and SRP, further supporting chl functional customization.

The role of the stem chl level in the rate of stem photosynthesis is not completely clear, in particular with the differences between SPC and SRP stems. On one hand, comparison between stems of different deciduous and evergreen trees found a correlation between the mass-based gross photosynthesis rate and the chl concentration [SRP-type: (Berveiller et al., 2007)]. Others found no correlation between the two [SRP and SPC-type stems: (Ávila et al., 2014)]. Indeed, the range of chl levels in stems of different families is quite wide and does not necessarily distinguish between SRP (0.72–4.8 mg/dm^2^) and SPC species (2–13 mg/dm^2^) (Ávila et al., 2014). Even comparison between stems' chl levels (SRP type) from species of the same genus (Prunus) showed large variation (Pilarski et al., 2007).

Chl levels of *P. dulcis* and *P. arabica* 1^*st*^-year stems were significantly lower than the levels that have been previously documented for other members of the Prunus family (Cherry or plum, 1.6–2.2 mg/dm^2^) (Pilarski et al., 2007). Interestingly, chl levels in the 1^*st*^-year stems of *P. arabica* were higher by 2.8-fold from 1^*st*^-year stems of *P. dulcis*, reaching 1.4 mg/dm^2^. Both almond species showed a small decrease (yet not significant) in stem chl concentration with stem age. Indeed, previous studies have questioned the effect of stem age on stem chl content, yet the results are not conclusive. While few studies showed a reduction in stem chl concentration with stem age (Pfanz et al., 2002), others showed contradicting results (Pilarski et al., 2007). The small reduction in stem chl content observed here (1^*st*^ to the 2^*nd*^ year; Figure 3A) correlates with the decline in stem stomatal density (Figure 2A), which is probably due to the growth and expansion of the stems, and the result of a “dilution” effect.

Stems and leaves are anatomically different. Nevertheless, previous studies have compared stem and leaf chl content based on their projected area. It has been shown that SRP-type young twigs can reach between 30 and 70% of leaf chl content (Kharouk et al., 1995; Solhaug et al., 1995; Schmidt and Pfanz, 2000; Pfanz et al., 2002; Pilarski et al., 2007; Ávila et al., 2014). On the contrary, in some SPC-type plants, higher levels of chl were detected (Ávila et al., 2014), with no differences between stem and leaf chl content [*Justicia californica* (Tinoco-Ojanguren, 2008)]. In this study, we found some interesting results. While chl levels of *P. dulcis* stems and leaves were comparable to those of *P. arabica* leaves, chl levels of *P. arabica* stems were much higher. These results further support physiological differences between SPC (*P. arabica*) and SRP (*P. dulcis*) almond species.

In most plant species, reduction in the chl a/b ratio has been attributed to the physiological adaptation of the plant to low light intensities (Cernusak and Marshall, 2000; Aschan et al., 2001; Damesin, 2003; Wittmann and Pfanz, 2008; Yiotis et al., 2008). The thickening of the outer bark acts as a light barrier (Pfanz et al., 2002; Saveyn et al., 2010; Rosell et al., 2015). Indeed, it has been previously shown that the chl a/b ratio of barked beech stems is lower than its light-exposed leaves and resembles more the ratio of shade-adapted leaves (Tran, 2018). Low chl a/b ratios in leaves have been attributed to the shading effect, and in stems to the detraction in light penetration by the outer cork layers (Berveiller et al., 2007), and the depth of stem chlorophyllous cells (Pfanz et al., 2002). Interestingly, when chl a/b ratios were compared between the leaves and stems of each species, clear significant lower ratios were detected in *P. dulcis* stems but not in *P. arabica*. Furthermore, these relatively low chl a/b ratios were even lower in the 2^*nd*^-year stems of *P. dulcis* (Figures 3B,D). This result can be explained by the development of stem cork layers in *P. dulcis* in their 2^*nd*^ year, and the distribution of the chl in deeper stem layers, as discussed below.

Different studies have shown a diverse pattern of chl distribution among different species (Berveiller et al., 2007; Burrows and Connor, 2020). In stems of both SRP and SPC-type plants, carbon fixation occurs in the chlorenchyma, which is distributed in different stem tissue layers (Cannon and Knox, 1908; Langenfeld-Heyser, 1989; Yiotis et al., 2008; Ávila et al., 2014). In SRP-type species, chl was found in different cells and in different stem depths, varying from chlorenchyma beneath the epidermis or phellem, *via* the cortex, surrounding xylem vessels, and as deep as stem pith (Berveiller et al., 2007; Burrows and Connor, 2020). These chlorophyllous cells were suggested to play a role in CO_2_ refixation (Sun et al., 2003). Yet not all chlorophyllous cells play a role in photosynthesis, and, in some cases, chl formation occurs under extremely low light intensities below that needed for photosynthesis (Schaedle, 1975). On the other hand, in many SPC-type species, the greatest concentration of chlorophyllous tissue was detected in a narrow layer immediately beneath the epidermis (Gibson, 1983; Yiotis et al., 2006; Yiotis and George, 2011). It has been proposed that the thickness of the chlorenchyma correlates with light transmission into the different stem layers and, therefore, will be thicker in cork-less stems (i.e., SPC type) (Pfanz et al., 2002). In this study, a pronounced difference in the distribution of chl within the stem tissue was observed between the two almond species. While, in *P. dulcis*, the chlorophyllous cells were dispersed in the stem cortex under the cork layer (beneath the periderm), condensely located along the xylem rays, reaching inner stem tissues down till the pith (Figure 3E), in *P. arabica* stems, most of the chl was concentrated in a multi-layer palisade-like parenchyma, just below the epidermis (Figures 3E, 6). These differences further support the diverse stem photosynthetic type between the two almond species.

(c) *P. arabica stem has anatomical features characteristic to SPC*.

This study detected distinctive anatomical features in *P. arabica* stems, which were absent from *P. dulcis* stems. *P. arabica* stems are composed of a cork-less epidermis (Figures 1B, 4A–F), with a well-developed cuticle (Figure 4F), which is required for protection, in particular when the bark layer is missing. *P. arabica* stem epidermis was found to be rich in sunken stomata (Figures 2A,B, 4C,F), producing substomatal chambers that open to a chloroplasts-rich palisade-like parenchyma (Figures 4C–F). This feature enables efficient gas exchange between the atmosphere and the main photosynthetic tissue of the stem. Indeed, the thick layer of palisade-like chlorophyllous cells beneath the epidermis features most SPC-type species (Gibson, 1983; Osmond et al., 1987; Redondo-Gomez et al., 2005; Yiotis et al., 2006; Yiotis and George, 2011). The involvement of these cells in SPC-type photosynthesis has been previously compared to the leaf palisade mesophyll, which plays the main photosynthetic tissue within the leaf (Gibson, 1983; Yiotis et al., 2006; Yiotis and George, 2011). Interestingly, *P. arabica* stem CO_2_ assimilation levels were comparable to those of its leaves during early and mid-summer, while leaves were fully functional (Figures 6B,D).

**Figure 7:**
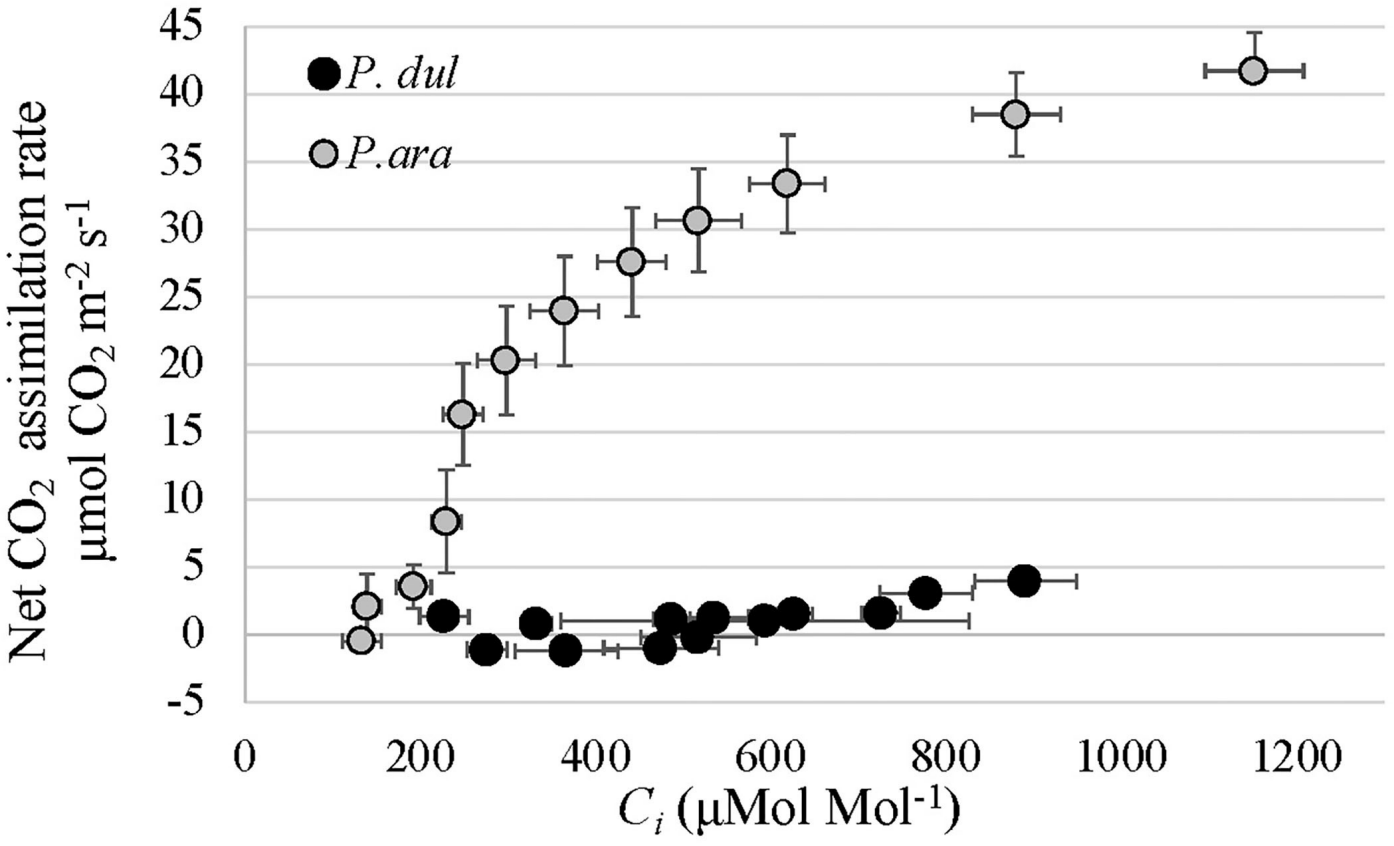
Response of net CO_2_ assimilation (*A*_*n*_) to increasing intercellular CO_2_ concentration (Ci) in 1^*st*^-year green stems of *P. arabica* (*P. ara*) and *P. dulcis* (*P. dul*). Each data point represents mean ± standard errors of 7 stems.

*P. dulcis* 1^*st*^-year stems show high similarity to *P. arabica* stems, including cuticle, sunken stomata within their epidermis layer, and a lack of periderm (Figures 4G–I). These anatomical features, presumably, enable efficient gas exchange with the atmosphere. Yet, their chlorenchyma cells were relatively small, rounded, and densely packed, unlike *P. arabica* “palisade-like” structure. The *A/Ci* curve revealed that the positive, yet extremely low CO_2_ assimilation levels of *P. dulcis* 1^*st*^-year stems (Figure 6B) were not due to a low Ci (intercellular CO_2_ concentration) (Figure 7). These data, together with the significant low levels of the Rubisco maximum carboxylation rate (Vc_max_), the maximum rate of electron transport (*J*_max_), and day respiration (Rd) (Table 1), further suggest that the anatomical difference in the fine structure of the photosynthetic apparatus affects the resistance along the CO_2_ diffusion pathway.

One of the unique anatomical features found in *P. arabica* but not in *P. dulcis* stems is the addition of five cortical bundles within the parenchyma layer (Figures 4A,D). Indeed, in certain plants, vascular bundles develop in the cortex in addition to the main vascular bundle of the stem. These vascular bundles are usually the vascular system of the leaf that connect to that of the stem and run along the stem cortex for some distance before its lower ends unite with the main vascular system (Metcalfe and Chalk, 1983). Although cortical bundles were found only in some but not all SPC-type species, they were suggested to play a role in photosynthetic sugar loading, allowing mass flow of phloem sap across the thick cortex (Mauseth, 2006; Rajput and Patil, 2016). Since the distance of the photosynthetic chlorenchyma in *P. arabica* is too far from the secondary phloem of the central cylinder, this unique cortical vascular system allows it to load sugars directly from the photosynthetic chlorenchyma and allocate them to other plant tissues.

### *P. arabica* and *P. dulcis* stems exhibit different photosynthetic performance

The results obtained from the chl fluorescence analysis (Figure 5), which provide insights into the photochemical and non-photochemical quenching characteristics of *P. arabica* and *P. dulcis* stems and leaves, are supported by their rates of CO_2_ assimilation (Figure 6). It has been shown earlier that the light-adapted quantum efficiency of PSII is well correlated with the CO_2_ assimilation rates in the stems of several deciduous tree species that carry out SRP (Wittmann and Pfanz, 2008). The stems of both species studied here conduct SRP (i.e., reassimilation from respiratory CO_2_); however, *P. arabica* also carries out CO_2_ assimilation of atmospheric CO_2_ *via* stem photosynthesis capability (SPC), as in leaves. The assimilation rates and transient NPQ induction of 1^*st*^-year *P. arabica* stems were considerably higher than *P. dulcis* 1^*st*^-year stems, supporting the rapid and higher quenching of Fm' in *P. arabica* (Figures 5C,F). Furthermore, considering *P. arabica* 1^*st*^-year stems vs. their leaves, the fluorescence quenching was quite similar, and NPQ was only slightly higher in leaves as compared to 1^*st*^-year stems. NPQ is a photoprotective mechanism essential for dissipating excess light energy (Ruban, 2016). Its activation is especially important under unfavorable environmental conditions when imbalances between the light reactions of photosynthesis and the downstream carbon fixation reactions occur. Rapid and higher NPQ induction in *P. arabica* stems can be attributed to its adaptation to hot and dry environments.

Interestingly, the steady-state fluorescence remains slightly higher in *P. arabica* leaves than in *P. dulcis* after the recovery from light (Figures 5E,H). This suggests lower fluorescence quenching by *P. arabica* leaves, which correlates with the significantly lower CO_2_ assimilation capacity of their leaves as compared to *P. dulcis* (Figure 6D).

### *P. arabica* SPC regulates transpiration and maintains high photosynthetic levels under elevated temperatures

Leaf transpiration has been proposed to play a role in plant cooling ability, preventing thermal damage (Schymanski et al., 2013), yet little is known about the direct effect of temperature on stomatal conductance regulation. Previous studies have revealed a range of responses, from temperature increases, which induced stomatal closure (Hamerlynck and Knapp, 1996; Mott and Peak, 2010; Lahr et al., 2015; Slot et al., 2016), to others, which induced stomatal opening (Feller, 2006; Marchin et al., 2016; Urban et al., 2017; Drake et al., 2018; Aparecido et al., 2020). This variability accounts for the different mechanisms in different species and the variability of the environmental conditions during the experiment (Schulze et al., 1973; Marchin et al., 2022). One of the main difficulties when studying plant physiological responses to temperature is the confusion between stomatal responses and the simultaneous change in water vapor-pressure deficit, which also affects stomatal conductance (Grossiord et al., 2020).

The effect of temperature increases on leaf transpiration and photosynthesis has been previously studied. Yet no research was done on the physiological responses of SPC and SRP-type plants to temperature. SPC-type species, including the wild almond *P. arabica*, are common in hot desert habitats and, therefore, more probable to comprise acclimation and adaptation mechanisms to confront the harsh environment. In this study, we evaluate specifically the effect of temperature increments (changed in a gas exchange chamber under constant VPD) on the physiological responses of *P. arabica* and *P. dulcis* leaves and stems.

Gas exchange results showed significantly high rates of transpiration and CO_2_ assimilation *via P. arabica* stems than *P. dulcis* stems (Figures 6A,B; Supplementary Figure S1). Interestingly, their rates were comparable in *P. arabica* stems and leaves (Transpiration: Figures 6A,C, Assimilation: Figure 6B) and responded similarly to temperature increment (Figures 6B,D). The physiological and anatomical similarity between *P. arabica* stems and leaves along with tree architecture (Figures 1–4), suggests them to play a similar role in tree carbon gain. On the other hand, in *P. dulcis* almond trees, stems showed negligible levels of transpiration and CO_2_ assimilation, while their leaves, which account for more than 90% of the aboveground surface area, showed significantly high CO_2_ assimilation rates.

Previous studies have found differences in stomatal conductance responses to elevated temperatures in different species (as previously discussed). Interestingly, both almond species, the wild SPC-type *P. arabica* (stems and leaves) and the commercial SRP-type *P. dulcis* (leaves), showed a similar decline in transpiration (Figures 6A,C), as well in their stomatal conductance (data not shown) in response to temperature increments. However, CO_2_ assimilation rates were slightly, yet not significantly reduced (Figures 6B,D). The lack of correlation between stomatal conductance and CO_2_ assimilation within the stem suggests Ci was not the limiting factor under these physiological conditions. These results further support conserved mechanisms, which preserve water under high temperatures, thus maintaining high CO_2_ assimilation rates, in both stems and leaves of these two almond species.

The anatomy of the 1^*st*^ and 2^*nd*^-year stems of *P. dulcis* was found to be significantly different. While 1^*st*^-year stems of *P. dulcis* were cork-less and contained stomata, no stomata could be detected on their 2^*nd*^-year stems, which developed a thick peridemal layer (Figures 2, 4). Surprisingly, the transpiration rates of both *P. dulcis* 1^*st*^ and 2^*nd*^-year stems showed similar levels and were dramatically lower than those of *P. arabica* stems. Previous studies, which showed that the permeability coefficients for peridermal water vapor diffusion resemble those of cuticles (Schönherr and Ziegler, 1980), suggest that the major part of transpiration detected in *P. dulcis* 1^*st*^ and 2^*nd*^-year stems is most likely passive and not regulated *via* the stomata.

The dependence of respiration on temperature has been previously studied in temperate fruit trees, showing a positive correlation between tissue temperature and its respiration rates (Sperling et al., 2019). Indeed, data from our previous study showed an increase in stem respiration rates of both *P. arabica* and *P. dulcis* 1^*st*^-year stems, in response to temperature increases, with no significant differences between the two species (for each measured temperature) (Figure 1F in Brukental et al., 2021). Indeed, some reduction (not significant) was observed in stem net CO_2_ assimilation rates, with increasing temperature, in each species (Figure 6B). The effect of temperature increases on the 2^*nd*^-year stems of *P. dulcis* was further supported by the significant decline in CO_2_ assimilation rates, reaching negative values. Since 2^*nd*^-year stems of *P. dulcis* lack stomata and are covered by a thick periderm, which blocks CO_2_ from entering and leaving the stem, it is most probable that the reduction in CO_2_ efflux that is observed with increasing temperatures attributes to elevation in respiration rates, yet further investigation is needed.

## Summary, importance, vision, and future perspectives

Here, we bring a detailed analysis of tree architecture, anatomical, and physiological comparison between the two almond species, the wild *P. arabica* and the cultivated *P. dulcis*. First- and 2^*nd*^-year stems of *P. arabica* showed anatomical and physiological features that support *P. arabica* to carry stem photosynthesis of SPC type, which include (1) a high stomatal density within the epidermis layer; (2) sunken stomata; (3) parenchyma composed of chl-rich palisade-like cells, as in leaf mesophyll; and (4) a delay of periderm formation (Gibson, 1983). Physiologically, (5) the performance of the light reaction centers of *P. arabica* stems is distinguishably different from those of *P. dulcis* stems and leaves; (6) its stems perform significantly high gross photosynthetic rates; and (7) its guard cells tightly regulate water loss in response to temperatures increments. On the other hand, the anatomical and physiological features of *P. dulcis*, as other cultivated deciduous fruit trees, support its stem photosynthesis to be of the SRP type. (1) Unlike *P. arabica* green stems, *P. dulcis* stems develop periderm; and (2) although *P. dulcis* young newly emerged stems possess few sunken stomata, with stem development, these stomata disappear under the thick bark layer (3) the chlorophyll level is significantly lower than those in *P. arabica* stems and distributes between various stem tissue layers. Physiologically, (4) its gross photosynthesis rates are relatively low and were not due to limiting Ci levels.

Considering climate change, the expected forecasts, and in light of the damage that already exists in crop indices, there is an urgent need for the development of new almond varieties that have an advantage in the environment of the future. Identification of unique traits among wild species that involve trees' resistance to harsh environments and implement them in breeding programs is a productive strategy that will provide us with new cultivars with physiological advantages. The unique trait of stem photosynthesis capability (SPC) of *P. arabica* holds various advantages in the face of climate change. This unique trait, which characterizes desert plants that live in extreme growing conditions and has been accredited to provide continuous SPC enables continuous photosynthetic activity even when leaves, the main photosynthetic organ, are absent (Nilsen, 1995; Ávila-Lovera et al., 2019). This additional energetic source of carbon plays an advantage for the plant to combat harsh growing environments. Furthermore, SPC also provides transpiration force and may increase the absorption of minerals from the soil (*via* mass flow) during the wintertime, when deciduous trees are leafless. During the winter season, carbon and minerals are needed for awakening from dormancy and to support flowering and fruit bearing as the energy stores of the tree are depleted. This unique feature may be of significant advantage in light of climate change, when chilling requirements are not fulfilled, and high temperatures increase the rate of respiration and utilization of the plant's energy store. Based on the results presented here, we expect that integration of the SPC trait into commercial cultivars will positively affect yield (Brestic et al., 2018). The effect of SPC on yield and water utilization is currently under investigation, with the aim of developing new hybrid cultivars well adapted for future climatic challenges.

## Data availability statement

The raw data supporting the conclusions of this article will be made available by the authors, without undue reservation.

## Author contributions

TT, HB, OS, ZA, and VT designed and conducted experiments. Surface area evaluation of the above ground tree parts was done by TT and HB. Stomatal imprint analyses were done by HB and ZA. Scanning electron microscopy (SEM) by TT and SG. Chlorophyll content measurement by TT. A chlorophyll autofluorescence confocal microscope by EB and TA-S. Histology by HZ and TA-S. Pulse-amplitude-modulated (PAM) chlorophyll *a* fluorescence by VT and DC. Gas exchange measurements by TT, HB, OS, and ZA. KH was in charge for planting, grafting, and maintaining the experimental orchard. DH was actively involved in the design of the research and provided suggestions throughout the research and wrote the manuscript with TA-S, with input from other authors. TA-S is the corresponding author, designed the experiments, and supervised the study. All the authors discussed and commented on the manuscript.

## Funding

This study was supported by the Leona M. and Harry B. Helmsley Charitable Trust.

## Conflict of interest

The authors declare that the research was conducted in the absence of any commercial or financial relationships that could be construed as a potential conflict of interest.

## Publisher's note

All claims expressed in this article are solely those of the authors and do not necessarily represent those of their affiliated organizations, or those of the publisher, the editors and the reviewers. Any product that may be evaluated in this article, or claim that may be made by its manufacturer, is not guaranteed or endorsed by the publisher.

## Abbreviations

A_n_: net CO_2_ assimilation rate
Chl: chlorophyll
Ci: intercellular CO_2_ concentration
E: transpiration rate
Fv/Fm: maximum photochemical efficiency of photosystem II
Gs: stomatal conductance
NPQ: non-photochemical quenching
SPC: stem photosynthetic capability
SRP: stem recycling photosynthesis
LG: linkage group
VPD: air vapor pressure deficit.
