# Supplementary material for: Physiological characterization of the wild almond Prunus arabica stem photosynthetic capability
Source: Front Plant Sci. 2022 Jul 29;13:941504. doi: 10.3389/fpls.2022.941504 (PMC9372545; doi:10.3389/fpls.2022.941504)
Supplement: Supplementary file 1 [file Data_Sheet_1.PDF]

Supplementary Material- Figures S1 and S2

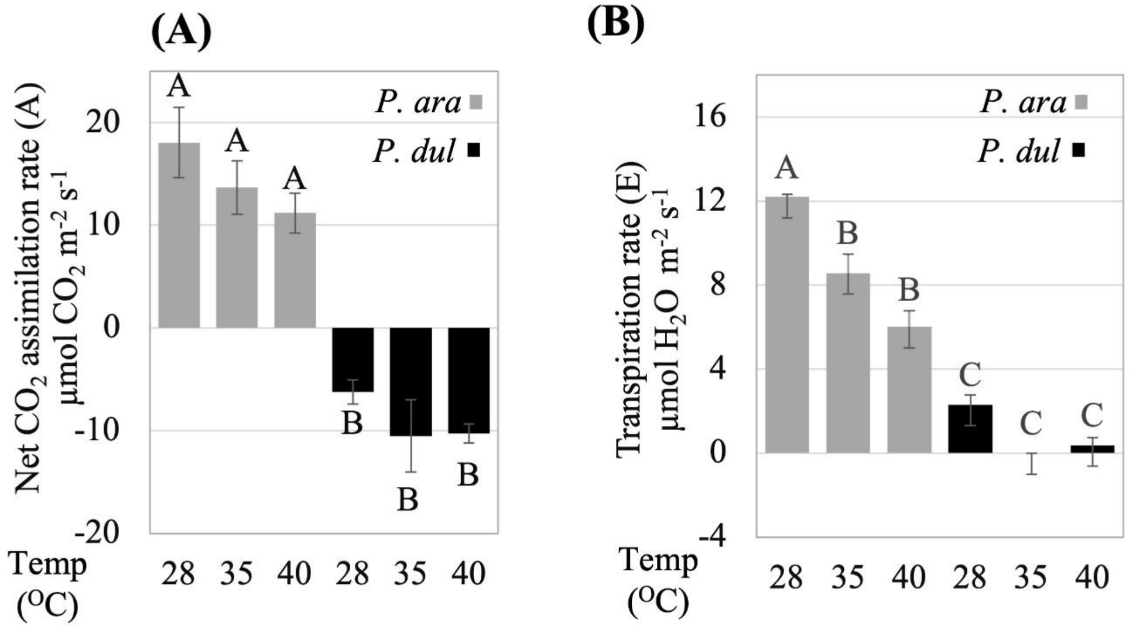

Supplementary Figure 1. Temperature extremes of 40°C modulate transpiration, while assimilation remain stable, in *P. arabica* 1st year stems. Gas exchange analyses of *P.arabica* (*P.ara*) and *P. dulcis* (*P. dul*) 1st year stems of young trees (3 years old) done during the summer season in response to temperature increments (28, 35, 40°C) and constant light (1200µE). Panels show **(A)** Net CO_2 assimilation rates and **(B)** transpiration rates. Full factorial analysis revealed significant variance in (a) almond variety ($P < 0.0001$ (b) Significant variance in almond variety and temperature ($P < 0.0001$ for each) and significant interactions between variety*temperature ($P = 0.0006$). Different uppercase letters above columns represent statistically significant difference (Tukey HSD, $P < 0.05$). Data are mean ± standard errors of 3 branches

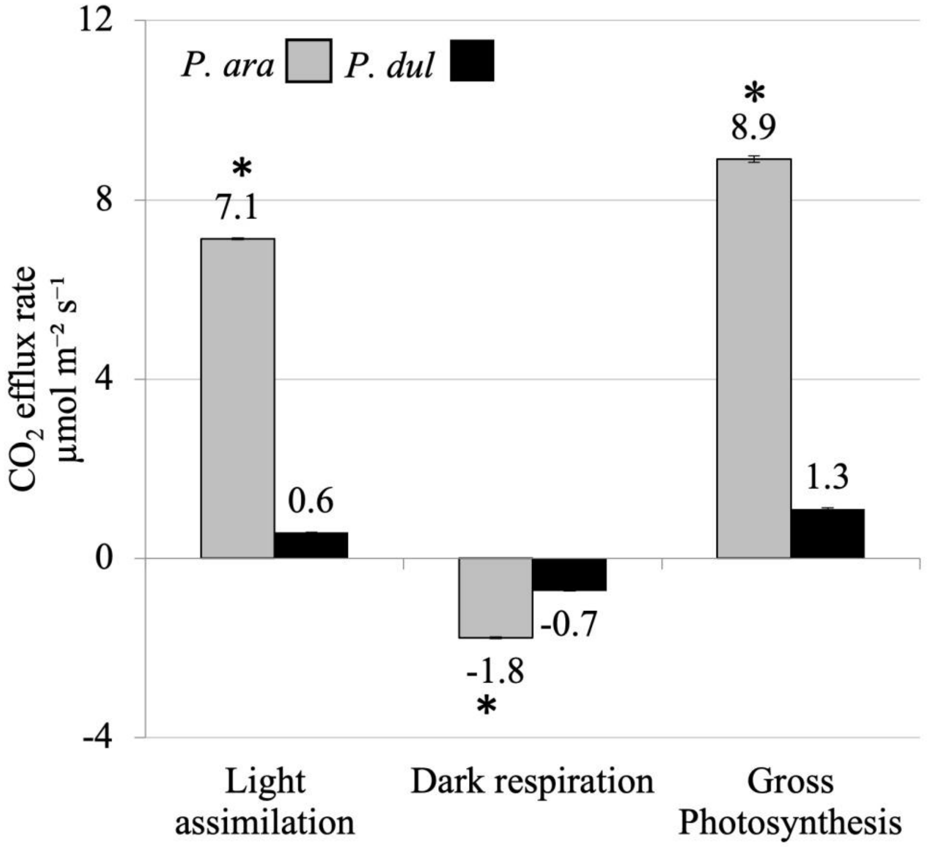

Supplementary Figure 2. Comparison of stem gross photosynthesis levels between *P. arabica* and *P. dulcis* 1st-year stems. Stem gas exchange analyses were done on 1st-year green stems of *P. arabica* and *P. dulcis* during the spring (February 2020) under light (1200µE) and dark conditions. Data present the CO_2 efflux rate in the light (i.e. light assimilation), in the dark (i.e. dark respiration), and the calculated stem gross photosynthesis rate ("light"-"dark" as described in (Cernusak and Marshall, 2000) . Data are mean ± standard errors of eight stems. The asterisks above the columns indicate statistical variability between species ($P < 0.05$ *t-test*). Fit model. $F < 0.0001$ for the whole model. P values < 0.0001, 0.0004, < 0.0001 for light assimilation, dark respiration, and gross photosynthesis, respectively.
